# Adolescents’ interactive electronic device use, sleep and mental health: a systematic review of prospective studies

**DOI:** 10.1111/jsr.13899

**Published:** 2023-04-07

**Authors:** Grace O. Dibben, Anne Martin, Colin B. Shore, Avril Johnstone, Christina McMellon, Victoria Palmer, Juliana Pugmire, Julie Riddell, Kathryn Skivington, Valerie Wells, Lisa McDaid, Sharon A. Simpson

**Affiliations:** ^1^ MRC/CSO Social and Public Health Sciences Unit University of Glasgow Glasgow UK; ^2^ Current Health Edinburgh UK; ^3^ Institute for Social Science Research The University of Queensland Brisbane Queensland Australia

**Keywords:** interventions, longitudinal studies, mobile devices, social media, teenagers, young people

## Abstract

Optimal sleep, both in terms of duration and quality, is important for adolescent health. However, young people's sleeping habits have worsened over recent years. Access to and use of interactive electronic devices (e.g., smartphones, tablets, portable gaming devices) and social media have become deep‐rooted elements of adolescents’ lives and are associated with poor sleep. Additionally, there is evidence of increases in poor mental health and well‐being disorders in adolescents; further linked to poor sleep. This review aimed to summarise the longitudinal and experimental evidence of the impact of device use on adolescents’ sleep and subsequent mental health. Nine electronic bibliographical databases were searched for this narrative systematic review in October 2022. Of 5779 identified unique records, 28 studies were selected for inclusion. A total of 26 studies examined the direct link between device use and sleep outcomes, and four reported the indirect link between device use and mental health, with sleep as a mediator. The methodological quality of the studies was generally poor. Results demonstrated that adverse implications of device use (i.e., overuse, problematic use, telepressure, and cyber‐victimisation) impacted sleep quality and duration; however, relationships with other types of device use were unclear. A small but consistent body of evidence showed sleep mediates the relationship between device use and mental health and well‐being in adolescents. Increasing our understanding of the complexities of device use, sleep, and mental health in adolescents are important contributions to the development of future interventions and guidelines to prevent or increase resilience to cyber‐bullying and ensure adequate sleep.

## 1 INTRODUCTION

### 1.1 Sleep problems in adolescents

Adequate sleep quantity and quality is imperative to the health and well‐being of young people (Dewald et al., 2010). Sleeping difficulties are common in adolescence (Inchley et al., 2020), and the biological and physiological changes occurring at this age can have an impact on sleep (Galván, 2020). The recommended sleep duration for adolescents, aged 13–18 years, is 8–10 h/day (Paruthi et al., 2016). However, there has been a detrimental shift in adolescent sleeping habits in recent decades, which means that many adolescents are not getting enough sleep (Hysing et al., 2015; Twenge et al., 2017). Conklin et al. (2019) argue that young people are suffering from chronic sleep disturbance, rather than deprivation, including issues such as insomnia and difficulty initiating or maintaining sleep.

### 1.2 The link between interactive electronic device (IEDs) use and sleep problems in adolescents

Over recent decades, IEDs such as smartphones and tablets have become essential to young people's development and ability to communicate with others (Vanden Abeele, 2016). Increases in IED use have run in parallel with the shift in adolescent sleeping habits, yet there is still relatively limited understanding of the implications that increased IED use might have on sleep (Lund et al., 2021).

For over two thirds of adolescents, the final activity engaged with before bed, at least three times per week, involves the use of an IED, and one‐third report using these devices in darkness prior to sleep (Kubiszewski et al., 2014; Mireku et al., 2019). Crucially, night‐time access to and use of IEDs has been associated with higher odds of poor‐quality sleep amongst young people (Carter et al., 2016; Hysing et al., 2013; Mireku et al., 2019).

Additionally, 93% of 13–17‐year‐olds have at least one social media account, and adolescents report engaging with such accounts for nearly 3 h/day on average (Barry et al., 2017). Studies have consistently found associations between social media use and sleep quality (Simsek & Tekgül, 2019; Twenge et al., 2017; Woods & Scott, 2016).

### 1.3 The link between sleep problems and mental health

Investigations into sleep problems in adolescents have highlighted their inextricable link with mental health outcomes (Owens et al., 2014; Scott & Woods, 2019). In the UK, the number of young people aged 5–15 years with a probable mental health disorder has increased over recent years to one in six (NHS Digital, 2020, NHS Digital, 2018). Going to bed later, spending less time asleep on weeknights, and oversleeping at weekends, has been shown to increase the likelihood of mood and anxiety disorders in adolescents (Zhang et al., 2017).

Some evidence points to a bi‐directional relationship between sleep and mental health (Cortese et al., 2013), whereby mental health disorders, such as depression and anxiety, are also predictors of inadequate sleep amongst young people (Shochat et al., 2014). However, this is contested; a well‐conducted meta‐analysis suggests sleep problems exist only as a precursor to mental health disorders, and that current evidence for such disorders being predictive of poor sleep is weak (Lovato & Gradisar, 2014). It appears that the ‘dominant pathway’ is from poor sleep to the occurrence of mental health disorders; and importantly, sleep problems can be treated, with positive effects on mental health (Freeman et al., 2020).

Both sleep disturbance and IED use have been identified as risk factors for depression (Lemola et al., 2015; Mireku et al., 2019; Thomee, 2018). Notably, Lemola et al. (2015) found sleep disturbance to be a partial mediator in the relationship between IED use and depressive symptoms; and highlight that most other studies consider sleep as a covariate rather than a mediator. Therefore, there is a need for further exploration of the potential mediating role of sleep in the relationship between IED use and mental health, and to use this information to inform interventions to improve sleep.

### 1.4 Rationale for the present study

Current advice on IED use is based on reviews of literature focused largely on television viewing, but nowadays adolescent IED use largely involves mobile devices (Scottish Government, 2017). Given the constant and rapid developments in IEDs and applications (apps), up‐to‐date research on the effects of IED use on sleep and mental health and well‐being is needed to keep up with the adoption of novel technologies. Literature reviews on this topic have also focused mainly on cross‐sectional studies, which are unable to show causal or temporal relationships (Carter et al., 2016; Lund et al., 2021; Shochat et al., 2014; Thomee, 2018; Yang et al., 2020). Furthermore, the direct relationship between IED use and mental health has been recently investigated in a meta‐analysis of 531 studies, demonstrating a small bi‐directional association, but the indirect link involving sleep was not explored (Shin et al., 2022). As data based on up‐to‐date IED preferences is becoming increasingly available, an updated systematic review is necessary to consider implications for advice on adolescents’ IED use.

### 1.5 Research questions (RQs)

The aim of this systematic review was to answer the following RQs:

to what extent is adolescents’ IED use associated with sleep outcomes?

what is the potential role of sleep as a mediator or mechanism between screen time and mental health and well‐being outcomes?

## 2 METHODS

The reporting of this study was guided by the Preferred Reporting of Systematic Reviews and Meta‐analysis (PRISMA) (Page et al., 2021). This review is an updated and more focused version of a previous rapid review commissioned by the Scottish Government (Martin et al., 2019). The published review protocol can be found using the following link: https://www.gla.ac.uk/schools/healthwellbeing/research/mrccsosocialandpublichealthsciencesunit/programmes/complexity/complexinterventions/screen_sleep_mentalhealth/#publications.

### 2.1 Literature search and eligibility criteria

Nine electronic bibliographic databases were searched in October 2022: Cumulative Index to Nursing and Allied Health Literature (CINAHL; Elton B. Stephens Co. [EBSCO]), Education Resources Information Center (ERIC; EBSCO), Excerpta Medica dataBASE (EMBASE; OVID), Medical Literature Analysis and Retrieval System Online (MEDLINE; OVID), PsycINFO (EBSCO), International Bibliography of the Social Sciences (IBSS; ProQuest), Applied Social Sciences Index and Abstracts (ASSIA; ProQuest), Social Science Citation Index (Web of Science), and Emerging Sources Citation Index (Web of Science). The key terms for the search strategies across the databases related to: (i) the population (adolescents), (ii) IEDs and related software (e.g., smartphone, social media, mobile apps, etc.), (iii) sleep outcomes (e.g., sleep duration, sleeplessness, night‐awakening etc.), and (iv) mental health. Subject headings and search functions were adapted for each database. The full search string for MEDLINE (OVID) is shown in Supplement Table S1 in Appendix S1. The literature search was limited to records available in English language only from 2007 onwards, as this was when the first commercial smartphone was released.

Eligibility criteria (based on the PICOS [participants, interventions, comparators, outcomes, and study design] approach) are detailed in Table 1.

**TABLE 1:** Inclusion/exclusion criteria

Criteria	Inclusion	Exclusion
Population	Young people with a mean age of 10–19 years. Studies including children aged <10 years or adults aged >19 years alongside young people were only included when results were reported for young people separately.	Clinical populations, i.e., samples based on having a specific condition/disorder/disease, e.g., insomnia, diabetes.
Exposures	Engagement with mobile (i.e., portable) interactive electronic devices (IED; e.g., smartphones, tablets, laptops) and software accessible through mobile IEDs (e.g., social media, games, websites, messaging applications), including studies that assessed the effectiveness of mobile applications (apps) or websites designed to improve sleep or mental health outcomes. Interventions with parents as agents of change with reports of adolescents’ mobile IED use were also included.	Studies which referred to screen time in general without specifying the device and/or specific use (e.g., an app). This was to ensure that we captured evidence on contemporary screen technology rather than older screen technology such as televisions.
Outcomes	Sleep – objectively assessed or subjectively reported indicators of sleep health and diagnosed sleep disorders^a^. Mental health and well‐being – indicators of absence or presence of emotional, psychological, and social well‐being assessed using validated psychometric questionnaires, and/or diagnosed mental disorder.	Studies where mental health outcomes were reported without reference to sleep outcomes.
Study design	Controlled interventions designs with or without randomisation^b^. Single‐group pre–post interventions, longitudinal studies, and repeated cross‐sectional studies. Systematic reviews including the aforementioned study designs were included and reviewed for eligible primary studies.	Cross‐sectional studies and systematic reviews of cross‐sectional studies.
Study setting	Only high‐income countries defined by the World Bank (https://data.worldbank.org/income‐level/high‐income) (Supplementary Table S2 in Appendix S1)	Low‐ or middle‐income countries (LMICs) were excluded due to the assumption that access to mobile IEDs is more limited in LMICs
Publication type	Peer‐reviewed journal articles of result reports.	Study protocols, conference abstracts, theses, dissertations, book chapters and any other form of non‐peer reviewed publications.

^a^ Due to the unclear reporting around the term ‘sleep quality’, this review used the term sleep quality in its broadest sense, whilst highlighting where possible differences in reported studies (e.g., sleep duration versus uninterrupted sleep).

^b^ Eligible intervention studies are included under the assumption that intervening in IED use to influence sleep outcomes indicates an association.

### 2.2 Study selection and data extraction

The study selection process was performed by a total of six reviewers (AJ, AM, GOD, JP, KS, JR, and CBS) using Endnote reference management software. One reviewer screened titles, abstracts, and full texts of all potentially relevant studies, and a second reviewer independently screened a randomly selected 10% of the articles. Disagreements were resolved through discussion or involvement of a third reviewer.

A standardised data extraction form in Microsoft Excel was developed and pilot tested. The following information was extracted: study design, country, sample size, population characteristics, details of exposure and outcome assessment, study results (direction, effect size, statistical significance). Data extraction for all included studies was performed by one reviewer (of AM, AJ, GOD, JP, JR or CBS) and cross‐checked for accuracy by a second reviewer (VP or GOD).

### 2.3 Study quality assessment

The Cochrane Collaboration tool for assessing risk of bias was used (Higgins et al., 2011). Five quality domains were assessed: (i) selection bias, (ii) performance bias (i.e., bias in assessment of the exposure), (iii) detection bias (bias in assessment of the outcome), (iv) attrition bias, and (v) selective reporting bias. Studies were judged to be of ‘high’, ‘unclear’ or ‘low’ risk of bias. One reviewer appraised the quality of all included studies (of AJ, GOD, JR, or CBS), which was cross‐checked for accuracy by another reviewer (of AM, GOD, or LM).

### 2.4 Data synthesis

Given the substantial heterogeneity across studies in terms of exposure assessment tools, outcome types and measurement, follow‐up, and effect estimates, a quantitative synthesis with meta‐analysis was deemed inapplicable. Hence, a narrative synthesis of the study findings was conducted and presented in line with the Synthesis without meta‐analysis guideline (Campbell et al., 2020).

Studies were thematically grouped into five main exposure categories based on the different type of IED use described in Table 2. Summary tables for each RQ (RQ1: IED use and relevant sleep outcomes; and RQ2: IED use and mental health outcomes) where sleep is a mediator were generated, mapping individual study results to each IED‐use category, and a vote‐counting approach was used to compare the number of results showing significantly positive or negative associations, or no association.

**TABLE 2:** Categories and subcategories of mobile interactive device use identified from the included studies

Type of IED use exposure (based on primary study author's description)	Definition
IED use Mobile phone use/ownership	The use of any type of IED at any time of day. The use of a mobile phone at any time of day, including smartphones/ownership of any IED, which would enable its use.
2 Social media use	Any interaction with a social media platform, whether actively or passively, at any time of day.
3 IED screen time/brightness	Time exposed to the screen light from any IED at any time of day.
4 Adverse implications of IED use IED overuse or problematic use Telepressure Cyber‐victimisation	Negative consequences following the use of IEDs. Addiction to, dependency to, or a maladaptive pattern of use of IEDs A psychological state in which the individual has a preoccupation with or urge to respond quickly to message‐based communications on IEDs. The experience of being victimised by others via the use of IEDs.
5 Smartphone applications as sleep aids	The use of smartphone applications to improve sleep and mental health outcomes.

Abbreviation: IED, interactive electronic device.

## 3 RESULTS

### 3.1 Literature search results

**FIGURE 1:**
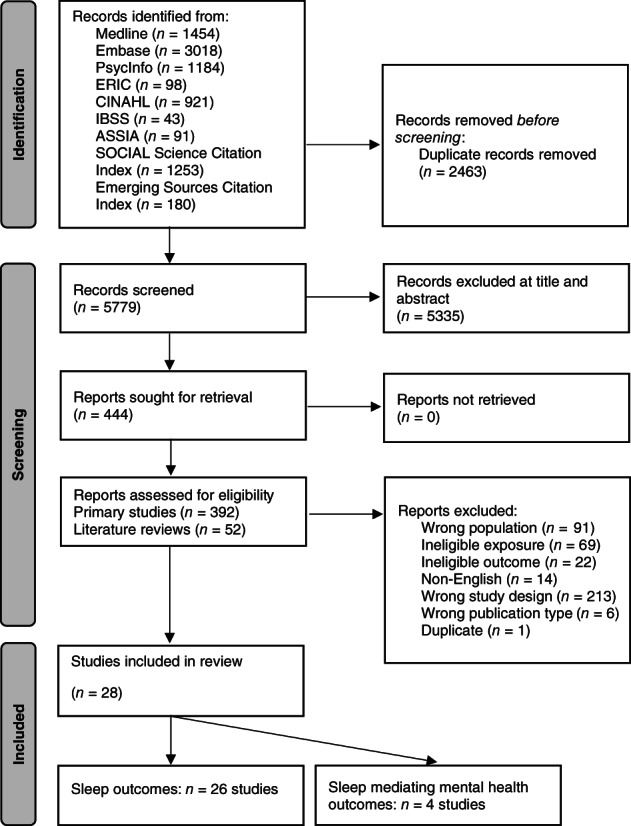
Preferred Reporting of Systematic Reviews and Meta‐analysis (PRISMA) flow diagram showing the literature search results

Figure 1 details the study selection process. In summary, database searches yielded 5779 unique records, of which a total of 28 studies met inclusion criteria. A total of 26 of these studies reported sleep outcomes and four examined the role of sleep as a mediator between IED use and mental health and well‐being outcomes (two studies reported findings relevant to both). References (numbered 1–28) for the included studies are provided in the supplementary files in Appendix S1.

### 3.2 Characteristics of included studies

Tables 3 and 4 present an overview of study characteristics, study exposure, mediator, direction of effects and risk‐of‐bias scores of reviewed papers. Detailed characteristics and results of reviewed studies are available in supplementary Tables S3 and S4 in Appendix S1.

**TABLE 3:** Identification, study characteristics, exposure, direction of effect and risk‐of‐bias scores of reviewed papers exploring interactive electronic device use and sleep outcomes

Paper identification number*	Lead author and year	Country	Study type, study characteristics	Exposure/intervention and outcome^a^	Direction of effect^a^	Description of effect	High risk‐of‐bias domains (out of five)
**IED use**							
1	Hamilton 2020	USA	Ecological momentary assessment	IED use (online gaming) and (I) sleep duration, (II) sleep timing	No effect (I, II)	No significant effects of gaming on (I) sleep duration or (II) sleep timing.	Two
2	McManus 2020	USA	Longitudinal, follow‐up: 3 months	IED use (video and interactive) and sleep quality	No effect (overall)	No association between IED use and lower sleep quality at follow‐up. Interactive screen time was associated with better sleep quality in males but remained poor in females	One
					Positive effect (males, interactive)		
3	Kemp 2020	Australia	Longitudinal, follow‐up duration: 2 years	IED use and time spent sleeping/napping	No effect	No significant association between IED use and time spent sleeping/napping	Zero
4	Poulain 2019	Germany	Longitudinal, average follow‐up: 12.4 months (range = 7.6–17.8 months)	IED use (A) computer/internet and (B) mobile phone; and (I) total sleep‐related problems, (II) bedtime problems, (III) sleep behaviour problems, and (IV) daytime sleepiness	Negative effect (AI, AII, AIV)	Higher levels of baseline computer/internet use was associated with an increase in (I) total sleep‐related problems, (II) bedtime problems, and (IV) daytime sleepiness at follow‐up, but not (III) sleep behaviour problems. Mobile phone use was not associated with an increase in any sleep related problems (I–IV) at follow‐up	Zero
					No effect (AIII, BI, BII, BIII, BIV)		
5	Gumport 2021	USA	Ecological momentary assessment	IED use and (I) TST, (II) bedtime, and (III) SOL	Negative effect (III)	Technology use associated with increased SOL No significant association between technology use and bedtime or TST	Zero
					No effect (I, II)		
6	Harbard 2016	Australia	Longitudinal cohort	Pre‐bedtime IED use: (A) phone and text messaging, (B) playing video games, (C) online chat/discussions, (D) online social networking, (E) web browsing for information, (F) streaming/downloading online media; and (I) bedtime, (II) rise time, (III) TST, and (IV) SOL	Negative effect (School – BII, BIII) (Vacation – BI, CII, CIV, DIV)	During school, video games were associated with later bedtime and shorter TST. No other screen‐based pre‐bedtime behaviours were associated with sleep variables. During vacation, video games were associated with later bedtime. Online chat was associated with later rise time and longer SOL. Social networking was associated with longer SOL. No other screen‐based pre‐bedtime behaviours were associated with sleep variables.	One
					No effect (School – AI, AII, AIII, AIV, BII, BIV, CI, CII, CIII, CIV, DI, DII, DIII, DIV, EI, EII, EIII, EIV, FI, FII, FIII, FIV) (Vacation –AI, AII, AIII, AIV, BII, BIII, BIV, CI, CIII, DI, DII, DIII, EI, EII, EIII, EIV, FI, FII, FIII, FIV)		
**Mobile phone use**							
7	Bartel 2019	Australia	Single arm pre–post intervention design, follow‐up: 2 weeks	Pre‐bed mobile phone use and (I) bedtime, (II) light out time, (III) SOL, (IV) TST, (V) sleep efficacy (Intervention—stop using phone 1 h before bed on school nights)	Positive intervention effect (II, IV, V). No intervention effect (I, III)	Participants turned lights off earlier and slept 21 min longer. Modest improvements in self‐assessed sleep efficacy. Participants did not go to bed earlier and did not fall asleep any quicker.	Three
8	Vernon 2018	Australia	Longitudinal cohort study, follow‐up: 1 and 2 years	Mobile phone use and sleep quality	Negative effect	Longer mobile phone use after bedtime was associated with lower sleep quality	Two
9	Foerster 2019	Switzerland	Prospective cohort study, follow up: 1 year	(A) Nocturnal mobile phone‐related nocturnal awakenings or (B) screen time and new onset of: (I) problems falling asleep, (II) restless sleep, (III) involuntary nocturnal awakenings, (IV) too early morning awakenings, (V) general sleep quality	Negative effect (AI, AII, BI)	Increased odds of developing sleep problems such as falling asleep and restless sleep in adolescents reporting large amounts of screen time or phone‐related awakenings. All other aspects of sleep problems were non‐significantly increased (III–IV)	Two
					No effect (AIII, AIV, AV, BII, BIII, BIV, BV)		
**Access to/ownership of IEDs**							
10	Schweizer 2017	Switzerland	Longitudinal cohort study, follow‐up: 2 years	Smartphone ownership and (I) sleep duration and (II) sleep problems	Negative effect (I, II)	Smartphone owners were significantly more likely to have shorter sleep duration than non‐owners The prevalence of sleep problems increased among new‐owners of smartphones to reach the prevalence observed amongst owners.	Three
**Social media use (SMU)**							
1	Hamilton 2020	USA	Ecological momentary assessment	SMU and (I) sleep duration, (II) sleep timing	No effect (I)	Those who used more social media did not have (I) shorter sleep, but (II) went to sleep later.	Two
					Negative effect (II)		
11	Garett 2018	USA	Longitudinal cohort study, follow‐up: 10 weeks	SMU: (A) tweet time of day (weekend or weekday) – (I) morning (II) afternoon, (III) evening, (IV) late night; (B) length of weekday tweet (short or long); and (C) weekday tweet emotional state (angry, fearful, loving, joyful or neutral); and sleep quality	Negative effect (weekday – AIV; short – BIV; and C – fearful)	More frequent use of twitter on weekday late nights associated with poor quality sleep, but tweeting more frequently on weekday evenings associated with better quality sleep. No observed differences at any weekend time. Shorter tweets on weekday late nights associated with poor sleep quality, and long tweets associated with better quality sleep. Tweets characterised by fear during weekdays associated with lower sleep quality. No other significant associations for length or emotion of tweets.	Two
					Positive effect (weekday – AIII; and long – BIII)		
					No effect (weekday – AI, AII; weekend – AI, AII, AIII, AIV; short – BI, BII, BIII; long – BI, BII, BIV; C – angry, C – loving, C – joyful, C – neutral)		
12	Vernon 2017	Australia	Longitudinal cohort study, follow‐up: 1 year and 2 years	Problematic SMU and sleep disruption	Negative effect	Increasingly problematic social networking site (Facebook, Myspace, Bebo) use associated with increased sleep disruption	Two
13	van der Schuur 2019	Netherlands	Longitudinal, Wave 1: November 2014, Wave 2: March 2015, Wave 3: June 2015	SMU (A) and SMS (B) and (I) sleep latency and (II) daytime sleepiness	Negative effect (AII, BI, BII, between‐person level)	At the between‐person level, over the three waves, longer sleep latency was associated with higher SMS, and increased daytime sleepiness was associated with higher SMU and SMS. At the within‐person level, neither SMU or SMS further increased sleep latency or daytime sleepiness over time	Two
					No effect (AI between‐person level; AI, AII, BI and BII, within person level)		
14	Maksniemi 2022	Finland	Longitudinal, follow‐up: 6 years	SMU and bedtime	No effect (overall)	No clear pattern between SMU and bedtime across adolescence.	Two
**IED screen time/brightness**							
15	Patte 2017	Canada	Longitudinal cohort study, follow‐up: 4 years	IED screen time ([A] talking on the telephone, [B] surfing the internet, or [C] texting, messaging or emailing) and sleep duration	No effect (A, B, C)	No longitudinal effect on sleep duration when students increased their screen use of any type	Two
16	Perrault 2019	Switzerland	Intervention (non‐RCT), Duration of intervention: 2 weeks	Sleep education workshop plus interventional phase in which participants asked to stop using screen devices after 9:00 p.m. on school nights. Outcomes: (I) light off time, (II) sleep onset time, (III) wake‐up time, (IV) out of bed time, (V) time in bed, (VI) total sleep period, (VII) TST, (VIII) sleep efficiency	Positive intervention effect (I, II, V, VI, VII)	Decreased evening screen time was associated with advanced light off time, sleep onset time, time in bed, and increased sleep duration	One
					No intervention effect (III, IV, VIII)		
17	Yoo 2020b	Korea	Comparative analysis between two longitudinal cohorts, separated by 3 years	Time spent playing video games and sleep duration	Negative effect	Time spent on playing games impacted on sleep duration, and had a greater impact on the more recent birth cohort	One
18	Heath 2014	Australia	Within‐subject controlled study	IED screen brightness (three conditions) and (I) pre‐sleep sleepiness, (II) SOL, (III) slow‐rolling eye movements, (iv) slow‐wave sleep, (v) rapid eye movement, (vi) morning functioning	No effect (I, II, III, IV, V, VI)	No differences in self‐assessed or objectively measured sleep outcomes between screen light conditions (bright, filtered short‐wavelength, and dim)	Zero
**Adverse implications of IED use**							
**IED overuse or problematic use**							
19	Lee 2017	Korea	Longitudinal, Wave 1 = 2011 Wave 2 = 2012 Wave 3 = 2013	Mobile phone addiction and (I) sleep quality, (II) sleep duration	Negative effect (I)	Increased mobile phone addiction was associated with a higher risk of poor sleep quality. No association between mobile addiction and sleep duration	One
					No effect (II)		
20	Yoo 2020a	Korea	Longitudinal, follow‐up duration: 6 years	Mobile phone dependency and sleep duration	No effect	Mobile phone dependency not significantly related to decreasing sleep duration trajectory	Two
17	Yoo 2020b	Korea	Comparative analysis between two longitudinal cohorts, separated by 3 years	Smartphone overuse and sleep duration	Negative effect	Smartphone overuse had a greater impact on sleep duration trajectories in the 2000 birth cohort compared to the 1997 cohort	One
21	Kojima 2019	Japan	Repeated cross‐sectional study, each year between 2014 and 2016	Problematic internet use and (I) bedtime or (II) sleepiness after wakening in the morning	Negative effect (I, II)	Significant positive association between problematic internet use and having a late bedtime (>12:00 a.m.). A significant positive association between problematic internet use and sleepiness after awakening in the morning	Zero
22	Chang 2022	Taiwan	Longitudinal cohort study, follow‐up 1 year	Smartphone addiction and (I) sleep quantity and (II) sleep quality	Negative effect (I, II)	An increase in smartphone addiction predicted the onset and persistence of inadequate sleep quantity. An increase in smartphone addiction predicted the onset and persistence of poor sleep quality	Zero
**Telepressure**							
23	Barber & Santuzzi, 2017	USA	Longitudinal, cohort study, follow‐up: 5–9 weeks	Telepressure and sleep hygiene	Negative effect	An increase in telepressure was associated with poorer sleep hygiene.	Three
**Cyber‐victimisation**							
24	Jose & Vierling 2018	New Zealand	Longitudinal cohort study, follow‐up: 1 year and 2 years	Cyber‐victimisation and sleep adequacy	Negative effect	More frequent incidences of cyber‐victimisation were associated with poorer levels of sleep adequacy at follow‐up	One
14	Patte 2017	Canada	Longitudinal cohort study, follow‐up: 4 years	Cyber‐victimisation and meeting sleep recommendations	Negative effect	Experiences of cyber‐victimisation led to reduced likelihood of meeting sleep recommendations	Two
25	Herge 2016	USA	Repeated cross‐sectional study, three time‐points, 6 weeks apart	Cyber‐victimisation and sleep problems: (I) excess sleep, (II) sleep deficit	Negative effect (I, II)	Cyber‐victimisation may contribute directly to adolescents’ sleep problems	Zero
22	Chang 2022	Taiwan	Longitudinal cohort study, follow‐up 1 year	Online harassment and (I) sleep quantity and (II) sleep quality	Negative effect (I, II)	An increase in online harassment predicted the onset and persistence of inadequate sleep quantity An increase in online harassment predicted the onset and persistence of poor sleep quality	Zero
**Smartphone applications as sleep aids**							
26	Werner‐Seidler 2019	Australia	Pilot study (single arm pre–post intervention), follow‐up: 6 weeks	Sleep education app for mild insomnia (intervention – Sleep Ninja App) Users received prompt 1 h before bedtime to commence pre‐bedtime routine and encouraged to stop using electronic devices. Outcomes: (I) insomnia, (II) sleep quality, (III) SOL, (IV) night‐time awakenings, (V) sleep refreshingness, (VI) use of sleep medication, (VII) TST, (VIII) time in bed, (IX) time in bed after final morning wake up, (X) habitual sleep efficiency	Positive intervention effect (I, II, III, IV, V, VII, IX, X)	Significantly improved insomnia symptoms, sleep quality, SOL, night‐time awakenings, sleep refreshingness, TST, time in bed after final morning wake up and habitual sleep efficiency. No improvements in use of sleep medication or time in bed.	Four
					No intervention effect (VI, VIII)		

Abbreviations: app, application; IED, interactive electronic device; RCT, randomised controlled trial; SMS, social media stress; SMU, social media use; SOL, sleep onset latency; TST, total sleep time.

^a^ A coding system has been applied to effect results where multiple relevant exposures or outcomes were reported. Exposures are coded by letter, and outcomes by a roman numeral, e.g., (A) mobile phone or (B) video game effects on (I) bedtime and (II) TST. A negative effect of mobile phone use on bedtime would be coded as (AI).

* Full references in supplementary files in Appendix S1.

**TABLE 4:** Identification, study characteristics, exposure, mediator, mediation effect and risk of bias score of reviewed papers exploring IED use and mental health, mediated by the impact of IED use on sleep

Paper identification number	Lead author and year	Country	Study type, study characteristics	Exposure/intervention and outcome	Mediator	Mental health/well‐being outcome	Mediation effect^a^	Description of sleep as a mediator between IED use and mental health	High risk‐of‐bias domains (out of five)
**IED use**									
8	Vernon 2018	Australia	Longitudinal cohort study, Follow‐up: 1 year	Night‐time mobile phone use	Sleep quality	(I) Depressed mood, (II), externalising behaviour, (III), self‐esteem, (IV) coping	Small to large mediation effect (I–IV)	Change in sleep behaviour mediated the effect of change in night‐time mobile phone use on subsequent change in all well‐being slopes. The indirect to total effect size ratios indicated small to large proportions of the original relation between changes in night‐time mobile phone use and changes in well‐being were explained by the indirect effect of changes in sleep behaviour (externalising behaviour: 33%, self‐esteem: 50%, coping: 60%, depressed mood: 73%)	One
**Social media use**									
12	Vernon 2017	Australia	Longitudinal cohort study, follow‐up: 2 years	Social media use	Sleep quality	(I) Depressed mood, (II) externalising behaviour	Small to large mediation effect (I, II)	53% of the association between increased social networking and increased depressed mood was explained by increased sleep disruptions. Change in sleep disruptions partially mediated the effect of change in social networking use on change in externalising behaviour, with the effect size ratio indicating only 13% was explained by the indirect effect.	Zero
27	Viner 2019	England	Longitudinal, Wave 1 = 2013 Wave 2 = 2014 Wave 3 = 2015	Social media use	Sleep adequacy	(I) Psychological distress, (II) life satisfaction, (III) happiness, (IV) anxiety	Large mediation effect in girls (I–IV) smaller mediation effect in boys (I)	Among boys, sleep accounted for 4.8% of the effect of very frequent social media use on later mental health. Among girls, sleep mediated the effect of very frequent social media use on later mental health, accounting for 17%. Among girls only, sleep largely mediated the effect of very frequent social media use on later life satisfaction (33.9%), happiness (22.3%) and anxiety (12%)	Two
**Adverse implications of IED use—Cyber‐victimisation**									
28	Kwon 2020	USA	Prospective longitudinal cohort study, follow up: 6 months	Cyber‐victimisation	Sleep quality	Depressive symptoms	Significant mediation effect	A significant indirect effect was identified where the mechanism of being depressed among adolescents who were cyber‐victimised was better explained by the mediational pathway of poor sleep quality. Proportion NR	One

Abbreviations: IED, interactive electronic device; NR, not reported.

^a^ Mediation effect sizes as described by authors.

The median (range) sample size was 837.5 (16–26,205). Seven studies were conducted in Europe (references 4, 8, 9, 12, 13, 15, 27), eight in North America (references 1, 2, 5, 10, 14, 23, 25, 28), eight in Oceania (references 3, 6, 7, 11, 16, 17, 24, 26), and five in Asia (references 18–22). Study designs included 19 longitudinal cohort studies (references 2–6, 8–13, 15, 19, 20, 22–24, 27, 28), one comparative analysis between two longitudinal cohorts (reference 17), two repeated cross‐sectional studies (references 21, 25), three single‐arm pre–post design interventional studies (references 7, 16, 26), two ecological momentary assessment studies (references 1, 5), and one within‐subject controlled laboratory study (reference 18). The median (range) follow‐up time across all studies was 12 months (1 day to 6 years). The median (range) of study mean age of participants was 14.8 (10–19) years, and the median (range) percentage of female participants across all studies was 52% (46%–83%).

Regarding exposures, 10 studies used a form of general ‘IED use’ (references 1–10), four studies used ‘IED screen time or brightness’ (references 14, 15, 17, 18), and six studies used ‘social media consumption’ (references 1, 11–14, 27). In all, 10 studies considered the adverse implications of IED use on sleep outcomes (references 15, 17, 19–25, 28), and one study considered the use of an IED delivered intervention to improve sleep (26). Studies used a variety of methods to measure sleep outcomes; 15 relied on self‐reporting through a questionnaire or survey (references 1, 4, 7, 10, 11, 13–15, 17, 19, 20, 22, 24, 27, 28), sleep diaries were used in three studies (references 3, 7, 16), and four studies used objective methods, such as actigraphy, to measure sleep outcomes (references 5, 6, 15, 18). In all, 13 studies measured sleep duration (references 1, 3, 5, 6, 10, 15–17, 19, 20, 22, 26, 27), 13 measured sleep hygiene (i.e., healthy sleep habits) (references 1, 5–9, 13, 14, 16, 18, 23, 26, 27), 12 measured sleep quality (references 2, 7–9, 11, 12, 16, 19, 22, 24, 26, 28), and six measured sleep‐related problems (references 4, 10, 16, 18, 25, 26).

Four studies examined the role of sleep as a mediator between IED use and mental health and well‐being outcomes. Mental health and well‐being outcomes were assessed using previously validated questionnaires or scales in all studies (references 8, 12, 27, 28). Mental health and well‐being outcomes included depressive symptoms or depressed mood (references 8, 12, 28), anxiety (reference 27), externalising behaviour (references 8, 12), psychological distress (reference 27), self‐esteem (reference 8), coping (reference 8), life satisfaction (reference 27), and happiness (reference 27).

### 3.3 Risk of bias of included studies

**FIGURE 2:**
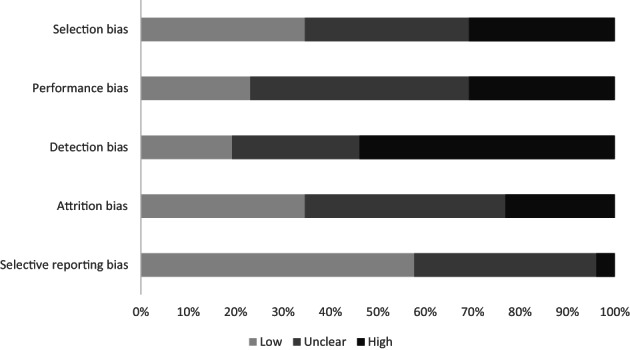
Risk of bias across studies assessing the association between interactive electronic devices use and sleep outcomes

Figure 2 summarises risk‐of‐bias assessments across studies exploring RQ1. In all, 20 of the 26 studies (77%) had at least one high‐risk‐of‐bias domain (Supplementary Table S5 in Appendix S1). There was a high risk of selection bias in eight (31%) studies, with convenience samples, poorly described recruitment strategies, small sample sizes, and limited generalisability across adolescents (references 1, 2, 6, 7, 11, 14, 23, 26). Eight (31%) studies had a high risk of performance bias, where studies either provided limited information regarding exposure measurement or used invalidated and/or adapted assessment tools, or blinding was poorly reported within interventional studies (references 8–10, 12, 15, 18, 23, 26). There was a high risk of detection bias for 14 (54%) studies, providing little to no information regarding measurement methods and psychometric properties of assessment tools for the self‐reported measurement of sleep outcomes (references 1, 7–15, 19, 20, 24, 26). Six (23%) studies had a high level of attrition and provided limited information on how this was handled in analyses (references 10, 13, 16, 19, 23, 26). Finally, selective reporting bias was judged to be unclear in 11 (42%) studies with no published protocols (references 8, 10–12, 15–17, 23, 24, 26) and high in only one (4%) study (reference 7).

**FIGURE 3:**
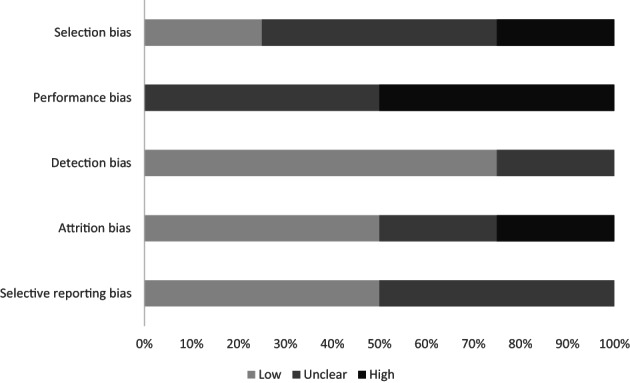
Risk of bias across studies assessing the potential role of sleep as mediator between screen time and mental health and well‐being outcomes.

Figure 3 summarises the risk‐of‐bias assessments across studies exploring RQ2. Three of the four (75%) studies had at least one high risk‐of‐bias domain (Supplementary Table S6 in Appendix S1). There was a high risk of selection bias in one study (25%) due to use of a convenience sample with limited generalisability (reference 28). A high risk of performance bias was detected in two (50%) studies (references 8, 27), with two (50%) studies judged as unclear due to lack of exposure measurement information or psychometric properties (references 12, 28). Detection bias was low in three (75%) studies that used validated questionnaires or scales to measure mental health and well‐being outcomes (references 8, 12, 27), and unclear in one (25%) study where a previously validated questionnaire was adapted by the authors (reference 28). There was a high risk of attrition bias in one (25%) study (reference 27), with high levels of drop out or exclusion from analysis. Selective reporting was generally adequate across studies with two (50%) low risk of bias (references 27, 28) and two (50%) unclear risk of bias studies (references 8, 12).

### 3.4 Association between interactive electronic media use and sleep

Table 3 presents an overview of the directions of effect with a full summary of relevant study findings across the 26 included studies presented in supplementary Table S3 in Appendix S1.

#### 3.4.1 Interactive electronic device use

The relationship between sleep outcomes and general IED use was assessed in six studies (*n* = 2006 participants) with inconsistent findings (references 1–6). Out of 63 individually reported outcomes across trials, only nine (14%) showed a significantly negative relationship, whereas the majority (53/63, 84%) demonstrated no association between IED use and sleep outcomes. In one study (reference 2) gender was identified as a moderator of the relationship between media use and sleep quality, where interactive screen time was in fact associated with better sleep quality in males.

##### Mobile phone use/ownership

One interventional study, and two longitudinal cohort studies examined the relationships between mobile phone use and sleep outcomes (references 7–9). Adolescents (*n* = 63) who participated in an intervention involving instructions to stop mobile phone use 1 h before bed stopped using mobile phones an average of 80 min earlier and participants also turned their bedroom lights off earlier and slept 21 min longer compared to baseline (reference 7). However, participants did not go to bed earlier and did not fall asleep any quicker (reference 7). Similarly, reported relationships reported amongst the longitudinal studies were conflicting (significant negative associations: four of 11 [36%], no significant associations: four of 11 [64%]). It is worth noting that the risk of bias was high amongst these studies. Assuming smartphone ownership implies use of the device, one study involving 591 adolescents showed that smartphone owners were significantly more likely to have shorter sleep duration than non‐owners, and the prevalence of sleeping problems increased over time among new owners to reach the prevalence among more long‐term smartphone owners (reference 10). However, this study was judged to have a high risk of bias in three out of the five domains.

#### 3.4.2 Social media use

Across five studies (*n* = 3014 participants) reporting associations between social media use and sleep, eight out of a total of 33 (24%) results demonstrated negative associations between social media use and sleep outcomes. This included more frequent use of Twitter, and shorter tweets on weekday late nights, and fearful tweets associated with poor quality sleep (reference 11), problematic use of social networking sites associated with increased sleep disruption (reference 12), and links between social media use or social media stress with later bedtimes (reference 1), sleep latency (reference 13), and daytime sleepiness (reference 13). Conversely, tweeting more frequently and longer tweets on weekday evenings were associated with better quality sleep (reference 11), and 23 (70%) of the reported results showed no association between social media and sleep.

#### 3.4.3 Interactive electronic device screen time/brightness

Four studies considered the effects of the duration of IED screen time or screen brightness on sleep outcomes, with conflicting results. Whilst one study with 26,205 participants found no longitudinal effect on sleep when students increased their screen use of any type (reference 15), another (*n* = 4335 participants) found that during school time, video games were associated with shorter total sleep time (reference 17). An intervention involving 569 adolescents with instructions to stop using screen devices after 9:00 p.m. on school evenings (Sunday to Thursday) for 2 weeks resulted in advanced light‐off time, sleep‐onset time, and increased sleep duration, but no improvements in wake‐up time, out of bed time, or sleep efficiency (reference 16).

In a laboratory study comparing three pre‐bedtime Apple iPad screen light conditions (bright, filtered short‐wavelength, and dim) in 16 adolescents, there were no differences in any self‐assessed or objectively measured sleep outcomes (reference 18).

#### 3.4.4 Adverse implications of IED use

##### Overuse or problematic use of IEDs

Five studies, all conducted in Asia (*n* = 13,278 participants), assessed the relationships between sleep and IED overuse or problematic use with six out of eight reported results (75%) indicating significantly negative associations. Increased mobile phone or smartphone addiction or overuse were associated with poorer sleep quality (references 19, 22) and sleep quantity (references 17, 22), and problematic internet use was associated with having a later bedtime and increased sleepiness after awakening in the morning (reference 21), whereas mobile phone dependency was not found to be associated with sleep duration (reference 20).

##### Telepressure

One study (*n* = 241 participants) found that an increase in telepressure (experiencing pressure to socially engage using a mobile phone) was associated with poorer sleep hygiene (reference 23).

##### Cyber‐victimisation

Four studies (*n* = 31,701 participants) examined the effects of cyber‐victimisation on sleep outcomes (references 14, 22, 24, 25). All six reported results across the four studies found that experiences of cyber‐victimisation were associated with poor sleep outcomes including sleep difficulties such as excess sleep and sleep deficiency (reference 25), and reduced likelihood of meeting sleep recommendations (references 14, 24), and that this association lasted up to 2 years after the cyber‐victimisation incidences occurred (reference 24).

#### 3.4.5 Smartphone applications as sleep aids

One study reported on the development and pilot testing of the Sleep Ninja smartphone app for adolescent insomnia symptoms (reference 26). The sleep training app included prompts to stop using electronic devices 1 h before bedtime. Preliminary results amongst the 50 participants showed improvements across eight of the 10 sleep‐related outcomes (insomnia symptoms, sleep quality, sleep onset latency, night‐time awakenings, sleep refreshingness, total sleep time, time in bed after final morning wake up, habitual sleep efficiency). However, no improvements were found in use of sleep medication or time in bed, and risk of bias was high in four of five domains.

### 3.5 Mediating role of sleep on mental health and well‐being

Table 3 presents an overview of the mediation effects of sleep on mental health and well‐being with a full summary of main findings presented in supplementary Table S4 in Appendix S1.

#### 3.5.1 Interactive electronic device use

One study involving 1101 adolescents found that changes in sleep quality mediated the relationship between changes in night‐time mobile phone use, and several indicators of adolescent well‐being including depressed mood, externalising behaviour, self‐esteem, and coping (reference 8), with small‐to‐large mediation effects.

#### 3.5.2 Social media use

Two studies (*n* = 13,740 participants) considered sleep as a mediator in the relationship between social media use and mental health and well‐being (references 12, 27). Poor sleep quality accounted for 53% of the association between increased social networking and increased depressed mood, and partially influenced (accounting for 13%) the association between social media use and externalising behaviour (13%) (reference 12). However, when considering alternative models in the opposite direction, for externalising behaviour, relationships seemed to run in both directions; problematic social media use led to externalising behaviour and vice versa, both mediated by sleep quality (reference 12). Similarly, inadequate sleep was shown to partially mediate the relationship between frequent use of social media and later mental health amongst boys and girls, and more largely mediate the effect on life satisfaction, happiness, and anxiety in girls only (reference 27).

#### 3.5.3 Adverse implications of IED use

In one study, whilst no direct association was found between cyber‐victimisation and depressive symptoms, a significant indirect effect was demonstrated, where adolescents who were cyber‐victimised were more likely to have poor sleep quality, which subsequently predicted increased depressive symptoms over time (reference 28).

## 4 DISCUSSION

### 4.1 Main findings

The aim of this systematic review was to summarise the published experimental and longitudinal evidence for IED use and its impacts on sleep and mental health and well‐being in adolescents. In order to manage the considerable heterogeneity and complexity in the measurement and reporting of IED use, we thematically grouped IED exposures into five categories (general IED use, social media, IED screen time/brightness, adverse implications of IED use, and smartphone applications as sleep aids). Across the different IED exposure categories and subcategories, the number of reported results supporting a direct and adverse relationship between IED use and sleep in adolescents ranged from 14% to 100%. Conversely, sleep quality and sleep quantity were consistently shown to mediate the relationship between IED exposures and mental health and well‐being outcomes including depressive symptoms, anxiety, externalising behaviour, self‐esteem, coping, life satisfaction, and happiness. However, these findings must be interpreted with caution, as most are based on poor quality studies with a high risk of bias. The reliance on self‐reported measures, the mixture of exposures, and outcomes measured, makes it difficult to draw conclusions beyond the individual studies.

#### 4.1.1 To what extent is adolescents’ IED use associated with sleep outcomes?

Despite identifying a total of 23 longitudinal and three experimental studies that examined this relationship, uncertainty remains regarding the extent to which IEDs affect sleep outcomes in adolescents. Across the different IED exposure categories, evidence supporting a detrimental relationship between IED use and sleep outcomes (including quantity, quality [in its broadest sense], and variability) was largely inconsistent. However, there was consistency in the direction of effects for the category of adverse implications of IED use, suggesting that IED overuse or problematic use, telepressure, and cyber‐victimisation appear to directly influence both sleep quantity and quality in adolescents. This finding supports a recent review that focused specifically on relationships between problematic smartphone use and sleep among adolescents, and also tentatively concluded weak‐to‐moderate cross‐sectional correlations (Mac Carthaigh et al., 2020).

Other research further suggests that it may not simply be the ‘screen time’ but specific effects of IED use and social media that impacts sleep, compared to more passive screen‐based activities such as television watching (Hale & Guan, 2015). Social media inherently has more personally relevant information than gaming or viewing content (Nesi et al., 2018). Additionally, social feedback and one's standing in a peer network are important in adolescence (Nelson et al., 2005). However, while there are rewarding components of social media use (e.g., positive comments, ‘likes’, new followers), there are associated negative impacts (e.g. negative comments, absence of feedback, cyber‐bullying) (Shapiro & Margolin, 2014). The psychophysiological arousal of social media and IED use means it may be more difficult to disengage from and confers risk of later sleep timing and more variable sleep schedules (Hamilton et al., 2020).

In some respects, our findings of inconsistency across the other IED exposure categories contradict other reviews. Both Carter et al. (2016) and Lund et al. (2021) concluded stronger and more consistent associations between electronic media device use and both sleep duration and quality outcomes; however, both based their conclusions completely or heavily (respectively) on cross‐sectional evidence. We chose to exclude cross‐sectional studies due to their primary limitation in determining temporal effects or causality. Nevertheless, much like these two previous reviews, the included evidence in our present review is limited by a high risk of bias and high heterogeneity, which makes it difficult to combine and summarise results to draw strong conclusions.

Proposed mechanisms include the displacement or replacement of sleep by IED use and that IED use increases mental and emotional arousal, which could disrupt sleep (Cain & Gradisar, 2010). Physiologically, melatonin production increases in the evening in response to decreasing ambient light levels. However, IED use involves increased light exposure from displays that delays melatonin release, thus sleep onset may be inhibited. We identified one study that compared Apple iPad screen light conditions (bright, filtered short‐wavelength, and dim), concluding no differences in self‐assessed or objectively measured sleep outcomes (Heath, 2014). However, they do not exclude that a longer duration of use might have an impact. Therefore, further research should consider both screen brightness and length of exposure to reliably draw inferences. Comprehensive investigation into effect modifiers or mechanisms such as gender, socioeconomic status, parental influence or restriction, screen brightness and length of exposure would provide a more thorough understanding of the potential longitudinal associations.

#### 4.1.2 What is the potential role of sleep as mediator or mechanism between screen time and mental health and well‐being outcomes?

There is an abundance of evidence supporting associations between IED use or social media and adolescent mental health and well‐being (Keles et al., 2019; Shannon et al., 2022; Shin et al., 2022); however, it has been argued that sleep is amongst one of the largest contributing factors to adolescent well‐being (Gireesh et al., 2018). We believe this to be the first review to specifically consider the role of sleep as a mediator or mechanism between IED use and mental health and well‐being outcomes. We identified a small, but consistent body of evidence suggesting that changes in sleep behaviour acts as a mediator in the relationship between changing IED use and both mental health and well‐being outcomes.

Poor sleep quality has been associated with lower competencies in control and perception of one's emotions (Brand et al., 2016), alongside an impaired ability for behavioural and emotional regulation in youth (Clarke & Harvey, 2012). Coupled with changing peer dynamics within adolescents’ social and peer group makeup (reference 8), it may well be that IED use provides the conduit to impact sleep as a gateway for problematic social media use and cyber‐bullying, leading to ruminating thoughts and fear or anxiety throughout the night, especially when it occurs in the hours before bed (reference 28).

Interestingly, a small number of included studies observed gender differences in the relationships between IED use, sleep, and mental health. One study found that displacement of sleep mediated the relationship to a much smaller degree in boys compared to girls (reference 27). Similarly, in another included study the magnitude of the relationship between mobile phone addiction and sleep quality was greater in girls (reference 19), and in a third study gender was found to moderate the effect of IED screen time on sleep quality, where interactive IED time was associated with better sleep quality in males, but poorer sleep quality in females (reference 2). Boys and girls have been shown to engage in electronic media differently, with boys spending more time gaming, and girls spending more time on smartphones and social media (Twenge & Martin, 2020). Further suggesting that the type of IED use (e.g., passive versus active, communication‐based) could be important, as well as gender differential media use and underlying mechanisms, which warrants further exploration.

### 4.2 Future research recommendations

We identified three interventional studies that targeted pre‐bedtime IED use, with modest but promising results, including improvements in sleep duration, sleep quality, and sleep onset latency. However, results of these studies are limited by their respective study designs, where none are based on the ‘gold standard’ of randomised controlled intervention. Therefore, we did not give them any more weight than other study designs. Future interventions including education programmes to increase awareness of the potential risks of problematic IED use, prevent, or increase resilience to cyber‐victimisation, and ensure healthy sleep should be considered. Ideally interventions should be tested within a fully powered randomised controlled trial. Additional considerations worthy of investigation include: (i) is there an impact from the nature of viewing screens up‐close compared to traditional screen viewing practises associated with television; (ii) does the use of second or third screens (e.g., television, laptop, and smartphone together) have increased impact on sleep; and (iii) does the nature of the content being engaged with impact the effects on sleep?

### 4.3 Strength and limitations of the review process

The strengths of this review include comprehensive and up‐to‐date literature searches across nine electronic databases, the inclusion of longitudinal and experimental studies, robust risk‐of‐bias assessments, and the categorisation of exposures to consider the different types and complexity of IED use. There are some limitations to this review including the exclusion of studies not published in English, which may have introduced language bias. Substantial heterogeneity across studies in terms of outcome and exposure measurement methods, different metrics reported, and effect estimates meant that we were unable to perform meta‐analysis. To synthesise the results, we applied a vote counting approach; however, this methodology has limitations. The magnitude of effects and study sample sizes are not accounted for, and studies that report multiple results (e.g., some studies reported up to six different, but relevant, exposures and up to 10 different sleep outcomes) are ultimately given more weight than those that report a single result. We were also unable to examine the potential presence of publication bias.

## 5 CONCLUSION

This systematic review summarises the current longitudinal and experimental evidence of the impact of IED use on adolescent's sleep and subsequent mental health. Across the 28 included studies results demonstrated that it may not be as simple to suggest that IED use alone is the key factor, and that adverse implications of IED use including overuse, problematic use, telepressure and cyber‐victimisation can have an impact on several sleep characteristics. Evidence suggests that sleep acts as a mediator in the relationship between IED use and mental health and well‐being in adolescents. The methodological quality of the studies was generally poor, so findings must be interpreted with caution. The risks of problematic IED use or overuse, especially before bed, and potential impacts on sleep and subsequent adolescent mental health and well‐being are important areas of focus, and greater awareness and understanding would enable the development of future interventions and policies.

## AUTHOR CONTRIBUTIONS

Valerie Wells conducted the searches. Avril Johnstone, Anne Martin, Colin B. Shore, Grace O. Dibben, Juliana Pugmire, Julie Riddell, and Kathryn Skivington screened titles and abstracts, and full texts. Anne Martin, Avril Johnstone, Colin B. Shore, Grace O. Dibben, Juliana Pugmire, and Julie Riddell performed data extraction and risk‐of‐bias assessments. Grace O. Dibben and Victoria Palmer cross checked data extraction and risk‐of‐bias assessments for accuracy. Grace O. Dibben performed data synthesis. Anne Martin, Colin B. Shore, and Grace O. Dibben, drafted the manuscript, and all authors provided critical revision and approved the version of the manuscript submitted for publication.

## CONFLICT OF INTEREST STATEMENT

Grace O. Dibben, Anne Martin, Colin B. Shore, Avril Johnstone, Christina McMellon, Victoria Palmer, Juliana Pugmire, Julie Riddell, Kathryn Skivington, Valerie Wells, Lisa McDaid, Sharon Anne Simpson have no conflicts of interest to declare.

## Supporting information


**Appendix S1.** Supplementary Information

## Data Availability

Data sharing is not applicable to this article as no new data were created or analyzed in this study.
