# Supplementary material for: Adolescents’ interactive electronic device use, sleep and mental health: a systematic review of prospective studies
Source: J Sleep Res. 2023 Apr 7;32(5):e13899. doi: 10.1111/jsr.13899 (PMC10909457; doi:10.1111/jsr.13899)
Supplement: Supplementary file 1 — Appendix S1. Supplementary Information [file JSR-32-e13899-s001.pdf]

Adolescents' interactive electronic device use, sleep and mental health: A systematic review of prospective studies

Table of Contents – Supplementary material

Supplementary Table 1: Ovid MEDLINE(R) without Revisions 1996 to January Week 3 2

Supplementary Table 2: List of high-income countries 7

Supplementary references for included studies 8

Supplementary Table 3. Summary of findings on the relationship between IED use and sleep outcomes 11

Supplementary Table 4. Summary of findings on the relationship between IED use and mental health, mediated by the impact of IED use on sleep 54

Supplementary Table 5. Risk of Bias rating for individual studies assessing sleep outcomes 63

Supplementary Table 6. Risk of Bias rating for individual studies assessing mental health outcomes mediated by sleep measures 64

Supplementary Table 1: Ovid MEDLINE(R) without Revisions 1996 to January Week 3

#	Searches
1	Adolescent/
2	"adolesc*".ab,ti.
3	boys.ab,ti.
4	girls.ab,ti.
5	"child*".ab,ti.
6	freshman.ab,ti.
7	freshmen.ab,ti.
8	"college student*".ab,ti.
9	"generation Y".ab,ti.
10	"Millennial*".ab,ti.
11	"Schoolchild*".ab,ti.
12	"Teen*".ab,ti.
13	"Undergraduate*".ab,ti.
14	"young people* ".ab,ti.
15	"youth*".ab,ti.
16	"young person* ".ab,ti.
17	1 or 2 or 3 or 4 or 5 or 6 or 7 or 8 or 9 or 10 or 11 or 12 or 13 or 14 or 15 or 16
18	Internet/
19	Online Social Networking/
20	Online social networking.ab,ti.
21	Social Media/
22	"social network*".ab,ti.
23	"SNSs".ab,ti.
24	"Social networking site* ".ab,ti.
25	"website*".ab,ti.
26	online.ab,ti.
27	Digital media.ab,ti.
28	electronic media.ab,ti.
29	"screen use".ab,ti.
30	"screen time".ab,ti.

#	Searches
31	facebook.ab,ti.
32	whatsapp.ab,ti.
33	skype.ab,ti.
34	"video streaming".ab,ti.
35	Buzzfeed.ab,ti.
36	iTunes.ab,ti.
37	Instagram.ab,ti.
38	Pintrest.ab,ti.
39	Twitter.ab,ti.
40	Tumblr.ab,ti.
41	"Chatroom*".ab,ti.
42	Cyber.ab,ti.
43	Snapchat.ab,ti.
44	Youtube.ab,ti.
45	Bebo.ab,ti.
46	Myspace.ab,ti.
47	"Blog*".ab,ti.
48	Selfie.ab,ti.
49	Sexting.ab,ti.
50	"instant messaging".ab,ti.
51	Texting.ab,ti.
52	"Vlog*".ab,ti.
53	"mobile phone*".ab,ti.
54	"mobile gaming".ab,ti.
55	cell phone.ab,ti.
56	Cell Phone/
57	cellular phone.ab,ti.
58	"smartphone*".ab,ti.
59	"smart phone*".ab,ti.
60	"laptop*".ab,ti.
61	ipad.ab,ti.
62	"computer tablet*".ab,ti.

#	Searches
63	"mobile tablet* ".ab,ti.
64	scrolling.ab,ti.
65	18 or 19 or 20 or 21 or 22 or 23 or 24 or 25 or 26 or 27 or 28 or 29 or 30 or 31 or 32 or 33 or 34 or 35 or 36 or 37 or 38 or 39 or 40 or 41 or 42 or 43 or 44 or 45 or 46 or 47 or 48 or 49 or 50 or 51 or 52 or 53 or 54 or 55 or 56 or 57 or 58 or 59 or 60 or 61 or 62 or 63 or 64
66	Sleep/
67	Sleep Deprivation/ or Sleep Hygiene/ or Sleep Disorders, Circadian Rhythm/ or Sleep/ or "Sleep Initiation and Maintenance Disorders"/ or Sleep Wake Disorders/
68	sleeplessness.ab,ti.
69	"sleep problem* ".ab,ti.
70	"sleep duration".ab,ti.
71	sleeping.ab,ti.
72	Sleep paralysis.ab,ti.
73	insomnia.ab,ti.
74	"sleep quality".ab,ti.
75	Sleep deprivation.ab,ti.
76	"Sleep disorder*".ab,ti.
77	"Sleep onset".ab,ti.
78	"sleep latency".ab,ti.
79	Parasomnia.ab,ti.
80	"Somatic problems".ab,ti.
81	"Circadian rhythm".ab,ti.
82	"Nightmare*".ab,ti.
83	Night awakening.ab,ti.
84	Wakefulness.ab,ti.
85	66 or 67 or 68 or 69 or 70 or 71 or 72 or 73 or 74 or 75 or 76 or 77 or 78 or 79 or 80 or 81 or 82 or 83 or 84
86	17 and 65 and 85
87	well-being.ab,ti.
88	wellbeing.ab,ti.
89	Mental Health/

#	Searches
90	mental disorder.ab,ti.
91	"mental disorder*".ab,ti.
92	"mental illness*".ab,ti.
93	Depression/
94	Anxiety/ or Anxiety Disorders/
95	Sadness/
96	Emotions/
97	"mood disorder*".ab,ti.
98	Mood Disorders/
99	Loneliness/
100	Loneliness.ab,ti.
101	isolation.ab,ti.
102	isolated.ab,ti.
103	Self Concept/
104	self-esteem.ab,ti.
105	resilient.ab,ti.
106	resilience.ab,ti.
107	"social anxiety".ab,ti.
108	FOMO.ab,ti.
109	"Fear of Missing Out".ab,ti.
110	"Vulnerabl*".ab,ti.
111	Stress.ab,ti.
112	Fatigue.ab,ti.
113	Tiredness.ab,ti.
114	Fatigue/
115	Suicide/
116	suicide.ab,ti.
117	Suicidal ideation.ab,ti.
118	self-harm.ab,ti.
119	Self-Injurious Behavior/
120	"Quality of Life"/
121	87 or 88 or 89 or 90 or 91 or 92 or 93 or 94 or 95 or 96 or 97 or 98 or 99 or 100 or 101 or 102 or 103 or 104 or 105 or 106 or 107 or 108 or 109 or

#	Searches
	110 or 111 or 112 or 113 or 114 or 115 or 116 or 117 or 118 or 119 or 120
122	86 and 121

Supplementary Table 2: List of high-income countries

Source: [accessed 08.06.2020]
https://datahelpdesk.worldbank.org/knowledgebase/articles/906519-world-bank-country-and-lending-groups

Aruba	Macao SAR, China
Andorra	St. Martin (French part)
United Arab Emirates	Monaco
Antigua and Barbuda	Malta
Australia	Northern Mariana Islands
Austria	New Caledonia
Belgium	Netherlands
Bahrain	Norway
Bahamas, The	New Zealand
Bermuda	Oman
Barbados	Panama
Brunei Darussalam	Palau
Canada	Poland
Switzerland	Puerto Rico
Channel Islands	Portugal
Chile	French Polynesia
Curacao	Qatar
Cayman Islands	Saudi Arabia
Cyprus	Singapore
Czech Republic	San Marino
Germany	Slovak Republic
Denmark	Slovenia
Spain	Sweden
Estonia	Sint Maarten (Dutch part)
Finland	Seychelles
France	Turks and Caicos Islands
Faroe Islands	Trinidad and Tobago
United Kingdom	Taiwan, China
Gibraltar	Uruguay
Greece	United States
Greenland	British Virgin Islands
Guam	Virgin Islands (U.S.)
Hong Kong SAR, China	
Croatia	
Hungary	
Isle of Man	
Ireland	
Iceland	
Israel	
Italy	
Japan	
St. Kitts and Nevis	
Korea, Rep.	
Kuwait	
Liechtenstein	
Lithuania	
Luxembourg	
Latvia	

Supplementary references for included studies

In order of paper ID

1. Hamilton, J. L., Chand, S., Reinhardt, L., Ladouceur, C. D., Silk, J. S., Moreno, M., Franzen, P. L., & Bylsma, L. M. (2020). Social media use predicts later sleep timing and greater sleep variability: An ecological momentary assessment study of youth at high and low familial risk for depression. *Journal of Adolescence, 83*, 122-130. https://doi.org/10.1016/j.adolescence.2020.07.009
2. McManus, B., Underhill, A., Mrug, S., Anthony, T., & Stavrinos, D. (2020). Gender moderates the relationship between media use and sleep quality. *Journal of Sleep Research, 30*(4), Article e13243. https://doi.org/10.1111/jsr.13243
3. Kemp, B. J., Parrish, A., & Cliff, D. P. (2020). 'Social screens' and 'the mainstream': longitudinal competitors of non-organized physical activity in the transition from childhood to adolescence. *International Journal of Behavioural Nutrition and Physical Activity, 17*(1), Article 5. https://doi.org/10.1186/s12966-019-0908-0
4. Poulain, T., Vogel, M., Buzek, T., Genueit, J., Hiemisch, A., & Kiess, W. (2019). Reciprocal longitudinal associations between adolescents' media consumption and sleep. *Behavioral Sleep Medicine, 17*(6), 763-777. https://doi.org/10.1080/15402002.2018.1491851
5. Gumport, N. B., Gasperetti, C. E., Silk, J. S., & Harvey, A. G. (2021). The impact of television, electronic games, and social technology use on sleep and health in adolescents with an evening circadian preference. *Journal of Youth and Adolescence, 50*, 2351-2362. https://doi.org/10.1007/s10964-021-01429-9
6. Harbard, E., Allen, N. B., Trinder, K., & Bei, B. (2016). What's keeping teenagers up? Prebedtime behaviors and actigraphy-assessed sleep over school and vacation. *Journal of Adolescent Health, 58*(4), 426-432. https://doi.org/10.1016/j.jadohealth.2015.12.011
7. Bartel, K., Scheeren, R., & Gradisar, M. (2019). Altering adolescents' pre-bedtime phone use to achieve better sleep health. *Health Communication*, *34*(4), 456-462. 2019;34(4):456-62. https://doi.org/10.1080/10410236.2017.1422099
8. Vernon, L., Modecki, K. L., & Barber, B. L. (2018). Mobile phones in the bedroom: trajectories of sleep habits and subsequent adolescent psychosocial development. *Child Development, 89*(1), 66-77. https://doi.org/10.1111/cdev.12836
9. Foerster, M., Henneke, A., Chetty-Mhlanga, S., & Roosli, M. (2019). Impact of adolescents' screen time and nocturnal mobile phone-related awakenings on sleep and general health symptoms: a prospective cohort study. *International Journal of Environmental Research and Public Health, 16*(3), Article 518. https://doi.org/10.3390/ijerph16030518
10. Schweizer, A., Berchtold, A., Barrense-Dias, Y., Akre, C., & Suris, J-C. (2017). Adolescents with a smartphone sleep less than their peers. *European Journal of Pediatrics, 176*(1), 131-136. https://doi.org/10.1007/s00431-016-2823-6
11. Garett, R., Liu, S., & Young, S. D. (2018). The relationship between social media use and sleep quality among undergraduate students. *Information, Communication & Society, 21*(2), 163-73. https://doi.org/10.1080/1369118x.2016.1266374
12. Vernon, L., Modecki, K. L., & Barber, B. L. (2017). Tracking effects of problematic social networking on adolescent psychopathology: the mediating role of sleep

disruptions. *Journal of Clinical Child & Adolescent Psychology, 46*(2), 269-83. https://doi.org/10.1080/15374416.2016.1188702
13. van der Schuur, W. A., Baumgartner, S. E., & Sumter, S. R. (2019). Social media use, social media stress, and sleep: examining cross-sectional and longitudinal relationships in adolescents. Health communication, 34(5), 552-559. https://doi.org/10.1080/10410236.2017.1422101
14. Maksniemi, E., Hietajarvi, L., Ketonen, E. E., Lonka, K., Puukko, K., & Salmela-Aro, K. (2022). Intraindividual associations between active social media use, exhaustion, and bedtime vary according to age – A longitudinal study across adolescence. *Journal of Adolescence, 94*, 401-414. https://doi.org/10.1002/jad.12033
15. Patte, K. A., Qian, W., & Leatherdale, S. T. (2018). Modifiable predictors of insufficient sleep durations: A longitudinal analysis of youth in the COMPASS study. *Preventive Medicine, 106*, 164-70. https://doi.org/10.1016/j.ypmed.2017.10.035
16. Perrault, A. A., Bayer, L., Peuvrier, M., Afyouni, A., Ghisletta, P., Brockmann, C., Spiridon, M., Vesely, S. H., Haller, D. M., Pichon, S., Perrig, S., Schwartz, S., & Sterpenich, V. (2019). Reducing the use of screen electronic devices in the evening is associated with improved sleep and daytime vigilance in adolescents. *Sleep, 42*(9), Article zsz125. https://doi.org/10.1093/sleep/zsz125
17. Yoo, C. (2020b). Cohort effects associated with reduced sleep duration in adolescents. *Sleep Medicine, 67*, 184-190. https://doi.org/10.1016/j.sleep.2019.10.025
18. Heath, M., Sutherland, C., Bartel, K., Gradisar, M., Williamson, P., Lovato, N., & Micic, G. Does one hour of bright or short-wavelength filtered tablet screenlight have a meaningful effect on adolescents' pre-bedtime alertness, sleep, and daytime functioning. *Chronobiology International, 31*(4), 496-505. https://doi.org/10.3109/07420528.2013.872121
19. Lee, J. E., Jang, S. I., Ju, Y. J., Kim, W., Lee, H. J., & Park, E. C. (2017). Relationship between mobile phone addiction and the incidence of poor and short sleep among Korean adolescents: a longitudinal survey of the Korean Children & Youth Panel Survey. *Journal of Korean Medical Science, 32*(7), 1166-1172. https://doi.org/10.3346/jkms.2017.32.7.1166
20. Yoo, C. (2020a). Sleep duration change and its associated factors during adolescence: a 6 year longitudinal study. *Child Indicators Research, 13*, 573-590. https://doi.org/10.1007/s12187-018-9615-7
21. Kojima, R., Sato, M., Akiyama, Y., Shinohara, R., Mizorogi, S., Suzuki, K., Yokomichi, H., & Yamagata, Z. (2019). Problematic internet use and its associations with health-related symptoms and lifestyle habits among rural Japanese adolescents. *Psychiatry and Clinical Neurosciences, 73*(1), 20-26. https://doi.org/10.1111/pcn.12791
22. Chang, F-C., Chiu, C-H., Chen, P-H., Chiang, J-T., Miao, N-F., Chuang, H-Y., Huang, W-Q., & Tseng, C-C. (2022). Smartphone addiction and victimization predicts sleep problems and depression among children. *Journal of Pediatric Nursing,* e24-e31. https://doi.org/10.1016/j.pedn.2022.01.009
23. Barber, L. K., & Santuzzi, A. M. (2017). Telepressure and college student employment: the costs of staying connected across social contexts. *Stress & Health, 33*(1), 14-23. https://doi.org/10.1002/smi.2668

24. Jose, P. E., Vierling, A. (2018). Cybervictimisation of adolescents predicts higher rumination, which in turn, predicts worse sleep over time. *Journal of Adolescence, 68,* 127-135. https://doi.org/10.1016/j.adolescence.2018.07.011
25. Herge, W. M., La Greca, A. M., & Chan, S. F. (2016). Adolescent peer victimization and physical health problems. *Journal of Pediatric Psychology, 41*(1), 15-27. https://doi.org/10.1093/jpepsy/jsv050
26. Werner-Seidler, A., Wong, Q., Johnston, L., O'Dea, B., Torok, M., & Christensen, H. (2019). Pilot evaluation of the Sleep Ninja: a smartphone application for adolescent insomnia symptoms. *BMJ Open, 9*(5), Article e026502. https://doi.org/10.1136/bmjopen-2018-026502
27. Viner, R. M., Gireesh, A., Stiglic, N., Hudson, L. D., Goddings, A-L., Ward, J. L., & Nicholls, D. E. (2019). Roles of cyberbullying, sleep, and physical activity in mediating the effects of social media use on mental health and wellbeing among young people in England: a secondary analysis of longitudinal data. *The Lancet. Child & Adolescent Health*, *3*, 685-696. https://doi.org/10.1016/S2352-4642(19)30186-5
28. Kwon, M., Seo, Y. S., Nickerson, A. B., Dickerson, S. S., Park, E., & Livingston, J. A. (2020). Sleep quality as a mediator of the relationship between cyber victimization and depression. *Journal of Nursing Scholarship, 52*(4), 416-425. https://doi.org/10.1111/jnu.12569

Supplementary Table 3. Summary of findings on the relationship between IED use and sleep outcomes

Reference	Study characteristics	Sample characteristics	Exposure/Intervention Description	Outcome description	Findings	Narrative findings
**Interactive electronic device (IED) use**						
Hamilton et al., 2020	USA<br><br>Ecological momentary assessment (EMA)	N=76<br><br>Age: 9-13<br>Mean age: 11.28<br><br>Sex: 46% female<br><br>Other: Participants had parental history of recurrent depression (n=35 high risk, n=41 low risk).	***Videogame use:*** 9-day EMA data collection (five weekdays and four weekend days).<br>Android smartphone provided, with custom app installed. Participants were to describe their current activity (from dropdown menu) when prompted by app.<br><br>Weekday prompts: delivered once in morning and twice during specified time frames (between 4:00PM-9:30PM).<br>Weekend prompts: delivered eight times a day at random times (between 10:00AM–10:00PM), but not more than once per 1.5 hours.	**Sleep duration and timing:** Participants asked two questions relating to sleep onset and offset;<br>1. About what time did you go to sleep last night?<br>2. About what time did you wake up this morning?<br><br>Sleep duration = difference between sleep onset/offset times.<br><br>Variability of sleep was calculated using each participant's standard deviation for sleep duration and onset over 9-day period.	*Multi-level modelling, with full information maximum likelihood.*<br><br>**Sleep timing:**<br>videogame use -0.01 (95%CI -0.04 to 0.01)<br>p=0.28<br><br>**Sleep duration:**<br>videogame use -0.01 (95%CI -0.01 to 0.03)<br>p=0.24	There were no significant main effects of gaming on sleep duration or timing
McManus et al., 2020	USA<br><br>Longitudinal	N=98<br><br>Age: 16	***Media usage:*** Adapted version of the Media Multitasking Index (MMI) (Ophir et al., 2019)	**Sleep quality:** 19 items on Pittsburgh Sleep Quality Index (PSQI) used to calculate 7 scales;<br>1. Subjective sleep quality	*Hierarchical multiple linear regression*<br>F=4.87	Video screen time did not predict lower sleep quality at 3-

	Follow-up: 3 months	Mean age: 16.27, SD 0.29<br><br>Sex: 52% female<br><br>Ethnicity: 65% Black/African American, 31% White/ Caucasian, and 4% more than one race or other (this 4% removed from analysis)	measured hours spent per week using a variety of media. Media included print media, TV, computer-based video, music, non-music audio, video/computer games, voice calls, IM, email, web surfing, and other computer based applications. Text messaging was added index for this study.<br><br>Two variables were created:<br>1. "Video" - Screen-based media with little-to-no communication involved, ie. TV, computer-based video and web browsing.<br>2. "Interactive" - Screen-based media which involves active usage or communication, ie. video games, IM, email and texting.	2. Sleep onset<br>3. Number of hours of actual sleep<br>4. Sleep efficiency<br>5. Sleep disturbances<br>6. Use of medication as sleep aids<br>7. Daytime functioning difficulties.<br><br>Scores on 7 scales ranged from 0-3, and summed to produce a global score ranging from 0-21. Global score of >5 indicated clinical levels of poor sleep quality.	R^2 = 0.36<br>p<0.01<br><br>**Video screen time:**<br>3 months -β= 0.06, p>0.05<br><br>**Interactive screen time:**<br>3 months - β= -0.24, p=0.06<br><br>**Male x video screen time:**<br>Baseline -β= -0.19<br>3 months – β= 0.05<br><br>**Male x interactive screen time:**<br>Baseline - β= 0.36<br>3 months - β= -0.71 p<0.01<br><br>**Inclusion of interactions in model:**<br>F = 4.93<br>r^2 change = 0.07<br>p<0.01	months. Marginal evidence suggesting an association of interactive screen time with lower sleep quality at 3-month follow-up.<br><br>Gender moderated the effect of interactive screen time on sleep quality 3 months later, with interactive screen time associated with better sleep quality in males, but remaining poorer in females. Compared to females, interactive components of screen time may lessen worsening sleep quality over time in males.
Kemp et al., 2020	Australia<br><br>Longitudinal	N=1043<br><br>Age: 11-13	***Media use:*** Participants asked to complete time use diaries (TUDs), in	**Sleeping/napping:** Time spent sleeping/napping, as reported in	**Social screens:** Time spent sleeping/napping	No significant change was observed for time

	Follow-up duration: 2 years	Baseline mean age: 10.9, SD 0.3<br><br>Sex: 49% female	both waves, to measure time they spent on non-organised physical activity (PA), and 13 potentially competing activities, over 24hr period.<br><br>Competing activities included, other PA, daily living activities, sleeping/napping, homework/study, school lessons, shopping, music for leisure, reading for leisure, electronic gaming, television/movies, verbal communication, texting/emailing/social media, and other internet use (eg. general internet browsing, internet shopping).<br><br>Participants were mailed a paper diary to use to record their activities the day before their home interview. Instructions were provided, and the diary was in an open ended format to allow	TUDs, was recorded at baseline and follow-up.	Mean change (95%CI): −15.5 (−33.5, 2.5) min/day p>0.05<br><br>**Mainstream:**<br>Time spent sleeping/napping Mean change (95%CI): −4.0 (−10.4, 2.4) min/day, p>0.05	spent sleeping/ napping in either segment at follow-up.

			use of own words. During the home interview, TUD data was entered by interviewers using a predetermined coding framework.			
Poulain et al., 2019	Germany<br><br>Longitudinal cohort study<br><br>Average follow-up: 12.4 months (range = 7.6–17.8 months)	N= 467<br><br>Age: 10-17 years Mean age at baseline: 13.0<br><br>Sex: 48% boys, 52% girls	***Media consumption:*** Participants were asked to indicate how many hours per day they usually spend using different screen-based media, including TV or video, computer and internet, and mobile phones).	**Sleep related problems:** Assessed using a Sleep Self-Report (SSR), completed by study participants. The SSR included three scales, each of which included multiple questions:<br><br>Scale 1: Bedtime problems; including 9 questions on where, when and how children go to bed and fall asleep<br><br>Scale 2: Sleep behaviour problems; including 6 questions on how children sleep at night.<br><br>Scale 3: Daytime sleepiness; including 3 questions on how children wake up and feel during the day.	*Linear regression*<br><br>**Computer/Internet:** Total sleep-related problems: (N = 423) - 1.67 (0.75 – 2.60), p< 0.001<br><br>Bedtime problems: (N = 444): 1.28 (0.76 – 1.80), p< 0.001<br><br>Sleep behaviour problems (N = 450): 0.14 (−0.21 – 0.49), p=0.459<br><br>Daytime sleepiness (N = 458): 0.32 (0.02 – 0.63), p=0.037<br><br>**Mobile phone:** Total sleep-related problems (N = 423):	High baseline levels of computer/internet consumption was associated with an increase in total sleep-related problems, problems at bedtime and daytime sleepiness at follow-up.<br><br>Mobile phone use was not associated with any sleep related problems at follow-up.

					−0.35 (−1.16 – 0.46), p=0.400<br><br>Bedtime problems (N = 444): −0.15 (−0.61 – 0.31), p=0.526<br><br>Sleep behaviour problems (N = 450): 0.06 (−0.25 – 0.36), p=0.717<br><br>Daytime sleepiness (N = 458): −0.01 (−0.27 – 0.25), p=0.939	
Gumport et al., 2021	USA<br><br>Ecological momentary assessment (EMA)<br><br>Follow-up: 1 week	N=176 (39 included in analysis)<br><br>Age range 10-14 years, mean age 14.77± 1.84<br><br>Sex: 57.95% female<br><br>Ethnicity: 15.34% Hispanic or Latino;	***Technology use***<br>Participants received EMA calls from a trained research assistant, twice on weekdays between 4 and 9pm and 4 times on weekend between 11am and 9pm. Technology use was assessed during the EMA call, participants responded to the question "at the moment the phone rang, what were you doing?" Responses were coded and technology use was	***Sleep – total sleep time, bedtime and sleep onset latency***<br>Assessed via wrist-worn actigraphy (Actiwatch Spectrum [Philips Respironics, Bend, Oregon, USA]) over 7 days. The main sleep window was the longest period of sleep identified by the scoring algorithm within a 24-hour window. Concurrently collected sleep diary data was used to adjust the sleep window as needed.	*Hierarchical linear modeling using maximum likelihood estimation*<br>*Models included participants' age, sex, previous night's sleep, Children's Morningness-Eveningness Preference Scale score, and if the night assessed was a weekday / weekend day as covariates.*	Technology use was associated with an increased sleep onset latency, but not with total sleep time or bedtime.

		84.66% Not Hispanic of Latino<br><br>Family income ($):<br>3.41% ≤20,000<br>11.93% 20,001-50,000<br>23.86% 50,001-100,000<br>57.95% 100,000<br>2.84% refused/missing	identified. The type of technology was categorized into one of three categories: television, electronic games (e.g. video games, computer games), or social uses (e.g. social media, texting)		**Total sleep time**<br>β=0.14<br>95% CI = -0.56 to 0.85, p=0.69<br><br>**Bedtime**<br>β = 0.65<br>95% CI = -0.67 to 0.28, p=0.42<br><br>**Sleep onset latency**<br>β = 0.65<br>95% CI =0.17 to 1.23, p=0.008	
Harbard et al., 2016	Australia<br><br>Longitudinal cohort<br><br>Follow-up duration: Last 2 weeks of school term, and second week of vacation.	N=146<br><br>Age: school years 10, 11 and 12 Mean age: 16.2 (SD 1.0)<br><br>52.7% female	***Screen use:*** Participants completed daily self-report measures on pre-bedtime behaviours (PBBs) and PSAcog (Presleep Arousal Scale). Items of IED use included:<br>1. Phone and text messaging.<br>2. Playing video games (including games on video-game consoles, computer, iPhone/iPod, mobile phone, or other portable gaming devices).<br>3. Online chat/discussions (chatroom, instant	***Bedtime, Risetime, Total Sleep Time (TST), Sleep onset latency (SOL):*** This study used comparable models of Actiwatch-2 and Actiwatch-64 (Mini Mitter, Bend, OR). Data were collected with 1-minute epochs and analysed based on "medium" threshold for sleep/wake detection in Actiware 5.5. Both actigraph models contained an "event marker" button for registering Bedtime (BT) and Risetime (RT).	*Regression analyses, controlled for chronotype*<br><br>**School days**<br>**Bedtime:**<br>Video games<br>β= 11.77 (95% CI 0.88-22.66), p<0.05<br>No other significant PBB associations<br><br>**Rise time:**<br>No significant associations between rise time and any PBBs	During School, video games were associated with later bedtime and shorter TST. No other screen-based pre-bedtime behaviours were associated with sleep variables.<br><br>During Vacation, video games were associated with later bedtime. Online chat was associated with

			messengers, including audio and video chats).<br>4. Online social networking (e.g. Facebook, Twitter, Myspace).<br>5. Web browsing for blogs, news, and information<br>6. Streaming/downloading online media (music, photos, videos).		**TST:**<br>Video games<br>β= -14.39 (95% CI -23.12 to -5.66), p<0.01<br>No other significant PBB associations<br><br>**SOL:**<br>No significant associations with any PBBs<br><br>**Vacation days Bedtime:**<br>Video games<br>β= 13.61 (95% CI 0.09-27.13), p<0.05<br>No other significant PBB associations<br><br>**Rise time:**<br>Online chat<br>β= 19.79, (CI: 4.70-34.88), p<0.05<br>No other significant PBB associations<br><br>**TST:**<br>No significant associations with any PBBs	later rise time and longer SOL. Social networking was associated with longer SOL. No other screen-based pre-bedtime behaviours were associated with sleep variables.

					**SOL:**<br>Online chat<br>β=3.23 (CI:0 .66-5.81), p<0.05<br><br>Social networking:<br>β= 3.11 (CI: 0.56-5.66), p<0.05<br>No other significant PBB associations	
Mobile phone use						
Bartel et al., 2019	Australia<br><br>Single arm pre-post intervention design<br><br>Follow-up: 2 weeks	N=98 (63 included in analysis)<br><br>Age: 14-18 years<br>Baseline mean age: 16.3 years<br><br>Sex: 83% female<br><br>Other: Android phone users only	***Pre-bed mobile phone use on school nights***: Adolescents given individualised phone stop times, 1 hour before bed for one school week. At the end of the baseline week, instructions were sent to participant email addresses detailing the time they should stop their mobile phone use, for the school week only (Sunday-Thursday). This time was 1 hour prior to their average baseline weekday bedtime.<br><br>Participants installed free screen On/Off Logger Lite' application which records when phone	*An online sleep diary used to collect sleep outcomes for two consecutive weeks; only weekday data were used.*<br><br>***Bedtime:*** Defined as going to bed, prior to light out time.<br><br>***Light out time:*** Defined as turning the light off with the intention of sleeping,<br>after going to bed; obtained from the sleep diary; unit = clock time.<br><br>***Sleep onset latency (SOL):***	**Bedtime:**<br>Baseline: 22:17 (SE 0:07)<br>Follow-up: 22:13 (SE 0:08)<br><br>Non-significant pre-post difference, F=0.46, p=0.50<br>Cohen's d = 0.06<br>**Light out time:**<br>Baseline: 22:57 (SE 0:07)<br>Follow-up: 22:40 (SE 0:08)<br><br>F=9.00, p=0.01<br>Cohen's d = 0.30<br><br>**SOL:**<br>Baseline: 21.0 min (SE2.2)	No significant change to bedtime was observed during the intervention week.<br><br>Adolescents stopped using their mobile phones earlier during the intervention week, turned off their lights earlier.<br><br>No significant change to SOL was observed during

			screen is turned on. App available to Android users only.	Defined as time taken to get to sleep after light out. Obtained from Sleep diary; unit = minutes.<br><br>***Total sleep time (TST)***:<br>Online sleep diary programme used an algorithm to calculate total sleep time; unit = hours: minutes.<br><br>***Sleep efficacy***:<br>3-item survey:<br>1. If their sleep during the intervention week was either 'better than usual', 'the same', or 'worse than usual'.<br>2. If they thought their sleep improved due to the intervention ('improved a bit', 'stayed the same', 'got worse').<br>3. If they thought the intervention was 'highly effective', 'somewhat effective', 'neither effective	Follow-up: 19.9 min (SE 1.9)<br><br>F=0.34, p=0.57<br>Cohen's d = 0.06<br><br>**TST:**<br>Baseline: 7:36 (SE 0:07)<br>Follow-up: 7:57 (SE 0:08)<br><br>F=7.98, p=0.01<br>Cohen's d = 0.34<br><br>**Sleep efficacy:**<br>N=29<br><br>45% stated intervention improved sleep a bit<br>45% sleep stayed the same<br>7% sleep became worse<br><br>7% reported the intervention to be highly effective<br>38% reported it to be somewhat effective	the intervention week.<br><br>Overall, during the intervention week, adolescents slept longer.<br><br>Modest improvements in self-assessed sleep efficacy.<br><br>Limited support for intervention effectiveness from participants

				nor ineffective', or 'ineffective'.	48% reported it to be neither effective nor ineffective<br>7% reported it to be ineffective	
Vernon et al., 2018	Australia<br><br>Longitudinal cohort study<br><br>Follow-up: 1 and 2 years	N=1101<br>Age: 13-16 years<br>Baseline mean age: 13.5 years<br><br>Sex: 57% female<br><br>Ethnicity: 56.9%Caucasian, 7.1% Asian, 2% Aboriginal or Torres Strait Islander, 21.9% other<br>Other: 44% from lower SES	***Night-Time Mobile Phone Use:*** Students were asked if they had a mobile phone. If answered yes, they were asked "At what time of the night do you usually send or receive messages and/or phone calls?"<br>6 response options: never text or phone after lights out; immediately after lights out; 10–11 p.m.; 11 p.m.–12 a.m.; 12–1 a.m.; 1–2 a.m.; 2–6 a.m.; at any time of the night.<br><br>Coded on 6-point scale (0-5) as 0 = no mobile phone, 1 = never text or phone after lights out, 2 = immediately after lights out, 3 = before midnight, 4 = after midnight, and 5 = at any time of the night	***Sleep quality:*** Assessed using a scale consisting of the mean of eight items drawn from School Sleep Habits Survey. The sleep scale tapped perceptions about sleep quality and behaviour during the previous 2 weeks.<br><br>Responses were 1 = never, 2 = once, 3 = twice, 4 = several times, and 5 = every day/night. Higher scores = lower sleep quality	*Zero-order correlation*<br><br>1 year follow-up: r=0.17, p<0.05<br><br>2 year follow-up: r=0.16, p<0.05	Both night-time mobile phone use and poor sleep behaviour underwent positive linear growth over time of intervention.<br><br>Longer mobile phone use after bedtime was associated with lower sleep quality
Foerster et al., 2019	Switzerland	N= 895	***Mobile phone use:*** Adolescents' mobile phone use was assessed	***Problems falling asleep; Restless sleep; Involuntary awakenings during night; Too early morning***	*Logistic regression analysis*<br>*Adjusted for age,*	Mobile phone-related nocturnal awakenings at

	Prospective cohort study<br><br>Follow-up: 1 year	Age: 10.4 – 17.0 years<br><br>Girls N=457; 57% Boys N=368; 43.6%	via hierarchically structured questionnaire items. Questions included participants' ownership of a mobile phone (yes/no), if they leave it turned on at night (4-point Likert scale), and frequency of nocturnal awakenings by calls or text messages (4 ordinal categories).<br><br>Objective mobile phone use records starting 6 months prior to baseline (BL) until the date of follow-up (FUP) were obtained from mobile phone operators, if participants gave additional informed consent. For example, daily number of calls.<br><br>The duration of daily tablet, computer (PC), laptop and television (TV) use was also assessed. Total screen time = sum of these durations, complemented by time using mobile phone.	***wakenings; General sleep quality:*** Four items from the Swiss Health Survey (Schweizerische Gesundheitsbefragung) enquired about:<br>1. Problems falling asleep<br>2. Restless sleep<br>3. Involuntary awakenings during night<br>4. Too early morning awakenings<br><br>Survey questions were answered on 4-point Likert scales without a specified time frame (never/seldom/sometimes/often).<br><br>A binary variable - general sleep quality - was defined in accordance with the Swiss Health Survey manual, and considered prevalent if at least one of the four items was answered in the highest category.	*sex, class level at baseline, nationality, school level, physical activity at follow-up smoking status at follow-up, alcohol consumption at follow-up, area of residence, education of parents, number of days between baseline/follow-up, body height difference between baseline/follow-up, daytime mobile phone use.*<br><br>**Mobile phone-related nocturnal awakenings**<br>**Problems falling asleep (N 1 = 461):**<br>Exposed/exposed OR = 3.44 (1.03–11.54)<br>Non exposed/exposed OR = 2.31 (0.87–6.09)	both timepoints increased the odds of developing problems falling asleep at follow-up.<br><br>Mobile-phone related nocturnal awakenings at both timepoints increased the odds of developing restless sleep at follow-up<br><br>All other aspects of sleep problems were non-significantly increased.

			For nocturnal awakenings, the exposed group consisted of all participants with at least one mobile-phone related nocturnal awakening (due to incoming mobile phone calls or text messages) per month.<br><br>For total screen time the exposed group = individual total screen time above median at baseline [180.8 min/day]; median at follow-up [173.6 min/day]		**Restless sleep (N = 652):**<br>Exposed/exposed OR = 5.39 (2.13–13.65)<br>Non exposed/exposed OR = 2.02 (0.82–4.93)<br><br>**Involuntary nocturnal awakenings (N = 686):**<br>Exposed/exposed OR = 1.82 (0.54–6.11)<br>Non exposed/exposed OR = 1.62 (0.58–4.54)<br><br>**Too early morning awakenings (N = 651):**<br>Exposed/exposed OR = 1.82 (0.76–4.39)<br>Non exposed/exposed OR = 1.00 (0.37–2.65)	

					**General sleep quality (N = 639):**<br>Exposed/exposed OR = 2.39 (0.79–7.22)<br>Non exposed/exposed OR = 3.69 (1.49–9.12)<br><br>**Total screen time**<br>**Problems falling asleep (N 1 = 455):**<br>Exposed/exposed OR = 2.35 (1.27–4.34)<br>Non exposed/exposed OR = 2.64 (1.33–5.26)<br><br>**Restless sleep (N = 637):**<br>Exposed/exposed= 1.27 (0.71–2.29)<br>Non exposed/exposed= 0.91 (0.45–1.82)<br><br>**Involuntary nocturnal awakenings (N = 672):**	High levels of total screen time at

					Exposed/exposed= 0.95 (0.50–1.83)<br>Non exposed/exposed= 0.66 (0.28–1.52)<br><br>**Too early morning awakenings (N = 637):**<br>Exposed/exposed= 1.43 (0.83–2.46)<br>Non exposed/exposed= 0.80 (0.40–1.62)<br><br>**General sleep quality (N = 630):**<br>Exposed/exposed= 1.06 (0.54–2.06)<br>Non exposed/exposed= 0.45 (0.17–1.18)	both time points significantly associated with new problems falling asleep.<br><br>High levels of total screen time at follow-up only associated with new problems falling asleep.<br><br>All other aspects of sleep problems were non-significantly increased.<br><br>In general, participants with constantly high media exposure at BL and FUP had the highest odds of various sleep problems.
Access to/ownership of IEDs						
Schweizer et al., 2017	Switzerland<br><br>Longitudinal cohort study	N=591<br><br>Age: range not reported	***Smartphone ownership:*** Assessed using an online questionnaire; YES/NO response; answers categorised into:	***Sleep duration:*** Participants indicated how many hours on average they slept during school days, and during	*One-way ANOVA*<br><br>**School days:**	Adolescents who owned a smartphone were significantly more likely to have

	Follow-up: 2 years	Baseline mean age: 14.3 years<br><br>Sex: 50% females<br><br>Nationality: 83.5% Swiss<br><br>Other: Socio Economic Status 5% below average, 38% above average	1. Owners (ownership at baseline and follow-up; n=383)<br>2. New owners (ownership at follow-up only, n=153)<br>3. Non-owners (those not owning a smartphone at any time-point; n=55).	weekends/vacation. Minutes are given on a decimal scale.<br><br>***Sleep problems***: Assessed by a single question: "Over the last six months, have you ever had sleep problems?"<br><br>There were five possible answers dichotomised as 'at least once a week' (at least once a week, most days) and 'others' (never, less than monthly, about once a month); yes = sleep problems.	Owners 7.28h (SD 0.09) vs Non-owners 8.00h (SD 0.20) p=0.002<br><br>Owners 7.28h (SD 0.09) vs new-owners 7.54h (SD 0.09) p=0.104<br><br>New-owners 7.54h (SD 0.09) vs Non-owners 8.00h (SD 0.20) p=0.075<br><br>**Weekend/vacation:**<br><br>Owner vs New-owner: p=0.10<br><br>Owner vs Non-Owner: p=0.94<br><br>New-owner vs non-owner: p=0.91<br><br>*Bivariate analysis comparing Owners vs New-owners vs Non-owners:*	shorter sleep duration than non-owners.<br><br>The prevalence of sleeping problems increased between T0 and T1 among new-owners of a smartphone to reach the prevalence observed among owners.

					**Baseline sleep problems [yes]:** p<0.001<br><br>Owners: 35.2%<br>New-Owners: 19.8%<br>Non-Owners: 15.4%<br><br>**Follow-up sleep problems [yes]:** p=0.49<br><br>Owners: 33.7%<br>New-owners: 33.6%<br>Non-Owners: 23.4%	
**Social media use**						
Hamilton et al., 2020	USA<br><br>Ecological momentary assessment (EMA)	N=76<br><br>Age: 9-13<br>Mean age: 11.28<br><br>Sex: 46% female<br><br>Other: Participants had parental history of recurrent depression (n=35 high risk, n=41 low risk).	***Media use frequency:*** 9-day EMA data collection (five weekdays and four weekend days).<br>Android smartphone provided, with custom app installed. Participants were to describe their current activity (from dropdown menu) when prompted by app.<br><br>Weekday prompts: delivered once in morning and twice during specified time frames	**Sleep duration and timing:** Participants asked two questions relating to sleep onset and offset;<br>1. About what time did you go to sleep last night?<br>2. About what time did you wake up this morning?<br><br>Sleep duration = difference between sleep onset/offset times.<br><br>Variability of sleep was calculated using each participant's standard deviation for sleep duration and onset over 9-day period.	*Multi-level modelling, with full information maximum likelihood.*<br><br>**Sleep timing:**<br>SM use 0.02 (95%CI 0.01 to 0.04)<br>p=0.01<br><br>Effect of days of SM use on sleep timing:<br>Est. = 0.17<br>SE = 0.06<br>p<0.01	Youth who used more SM went to sleep later, but did not have shorter sleep duration. These youth also had higher levels of variability in both sleep timing and sleep duration.

			(between 4:00PM-9:30PM).<br>Weekend prompts: delivered eight times a day at random times (between 10:00AM–10:00PM), but not more than once per 1.5 hours.		**Sleep duration:**<br>SM use -0.01 (95%CI -0.02 to 0.01)<br>p=0.22	
Garett et al., 2018	USA<br><br>Longitudinal cohort study<br><br>Follow-up: 10 weeks	N=197<br><br>Age: 17-20 years<br>Baseline mean age:<br>18.1 years<br><br>Sex: 60% female<br><br>Ethnicity: 29% Hispanic, 27% Asian, 22% White non-Hispanic, 12% black, 10% other<br><br>Other: Students had to be active Twitter users, tweeting at least three times a week.	***Twitter use***: All tweets and retweets were downloaded and categorised into five emotions: fear, anger, love, joy, or neutral, using machine learning model (a Naïve Bayes classifier).<br><br>The classifier used a bag-of words approach. Monograms that appeared in at least three tweets, bigrams that appeared in at least six tweets, and trigrams that appeared in at least three tweets were included.<br><br>Time of the day and weekday were also reported.	***Sleep quality***:<br>Assessed using a weekly survey (items not reported). Rating on a 5-point Likert scale (response options not reported).	*Generalised linear mixed model (adjusted for sex, ethnicity, academic major, tweets/week)*<br><br>**Weekday:**<br>Morning tweets β = 0.00 (SE 0.111), p>0.05<br>Afternoon tweets β = -0.12 (SE 0.102), p>0.05<br>Evening tweets β =0.189 (SE 0.097), p<0.05<br>Late night (2am-6am) tweets β = -0.937 (SE 0.352), p<0.01<br><br>**Weekend:**	Tweeting more frequently on weekday late nights was associated with lower sleep quality, however, tweeting more frequently on weekday evenings was associated with better quality sleep.<br><br>No observed differences at any weekend time.<br>Shorter tweets on weekday late nights associated with poor sleep quality, and long tweets associated with better quality sleep.

					Morning tweets β = 0.167 (SE 0.135), p>0.05<br>Afternoon tweets β =0.167 (SE 0.135), p>0.05<br>Evening tweets β = −0.117 (SE 0.08), p= >0.05 (value not reported)<br>Late night tweets β =-0.413 (SE 0.139), p >0.05 (value not reported)<br><br>Weekday tweet length – short<br>Morning tweets β = 0.017 (SE 0.084), p>0.05<br>Afternoon tweets β = 0.041 (SE 0.082), p>0.05<br>Evening tweets β =-0.117 (SE 0.08), p>0.05<br>Late night (2am-6am) tweets β = -0.413 (SE 0.139), p<0.01<br><br>Weekday tweet length – long	Tweets made on weekdays, which were categorised into the fear emotion were associated with lower sleep quality. No other significant associations for length or emotion of tweets.

					Morning tweets β = 0.066 (SE 0.084), p>0.05<br>Afternoon tweets β = -0.024 (SE 0.08), p>0.05<br>Evening tweets β = 0.197 (SE 0.087), p<0.05<br>Late night (2am-6am) tweets β = -0.025 (SE 0.137), p>0.05<br><br>**Emotions:**<br>Weekdays<br>Angry tweets β = -0.205 (SE 0.169), p>0.05<br><br>Fearful tweets β = -0.302 (SE 0.131), p<0.05<br><br>Loving tweets: β = 0.026 (SE 0.138), p>0.05<br><br>Joyful tweets: β = 0.105 (SE 0.128), p>0.05	

					Neutral tweets: β = -0.135 (SE 0.131). $p>0.05$	
Vernon, 2017	Australia<br><br>Longitudinal cohort study<br><br>Follow-up: 1 year and 2 years	N=874<br><br>Age: 12-18 years Baseline mean age: 14.4 years (SD not reported)<br><br>Sex: 59% female<br><br>Ethnicity: 57.2% Caucasian, 7.2% Asian, 1.6% Aboriginal or Torres Strait Islander, and 23.3% other	***Social media use:*** assessed using a problematic use of social networking scale. 4 items measured the degree to which adolescents invest emotionally in social networking:<br>1. “I prefer to spend time on Facebook/Myspace/ Bebo rather than attend social activities/ events”.<br>2. “I use Facebook/Myspace/Bebo as a way of making me feel good”.<br>3. “I get into arguments with other people about the amount of time I spend on Facebook/ Myspace/Bebo”.<br>4. “If I can't access Facebook/Myspace/Bebo, I feel moody and irritable”.	***Sleep quality***: Items, adapted from the School Sleep Habits Survey, asked:<br><br>During the during the previous 2 weeks, how often have you: “felt tired or sleepy during the day”; “had an extremely hard time falling asleep”; “had a good night’s sleep (reversed)”; “felt satisfied with your sleep” (reversed).<br>Response option were 1 (never), 2 (once), 3 (twice), 4 (several times), and 5 (every day/night). Higher scores = poorer sleep quality.	Bivariate correlation:<br><br>1 year: $r=0.34$, $p<0.01$<br><br>2 years: $r=0.26$, $p<0.01$	Increasingly problematic social networking site use predicted increases in sleep disruption.
van der Schuur et al., 2019	Netherlands<br><br>Longitudinal	N= 1,441 (longitudinal analysis)	***Social Media Use (SMU):*** SM use was measured using 12 items, including: (1) Facebook, (2)	***Sleep latency:*** Sleep latency was measured with the question: ‘On average, how long does it take before you fall asleep?’.	*Cross-lagged panel model*<br>**Sleep latency –**	At the between person level, no significant correlations found

	Wave 1: November 2014<br>Wave 2: March 2015<br>Wave 3: June 2015	Age: 11-15 years<br>Mean age = 12.61, SD age = 0.75<br><br>Sex: 51% boys	Facebook Messenger, (3) Instagram, (4) WhatsApp, (5) Snapchat, (6) Pinterest, (7) Twitter, (8) Vine, (9) Tumblr, (10) Telegram, (11) Google+, and (12) YouTube.<br><br>***Social media stress (SMS)***<br>SM stress measured using 10 items: 5 items on emotional response to SM use: have you (1) felt tensed or restless when you could not use social media; (2) felt disappointed when you did not get an immediate response if you posted something on social media; (3) continuously pondered about something that happened on social media; (4) felt tense or restless when you knew you received a social media message but could not look at it immediately; (5) felt disappointed when you had not received a message on social media;	***Daytime sleepiness:*** A Sleep Reduction Screening Questionnaire (SRSQ) examined the consequence of sleep reduction during daytime.<br><br>The SRSQ included nine items (e.g., 'I feel sleepy during the day', 'I am a person who does not get enough sleep', and 'I have enough energy during the day to do everything [reversed]').	**Between-person level:**<br>SM use b*=0.07, p=0.137<br>SM stress b*=0.19, p=0.003<br><br>**Within-person level:**<br>SMU → SLEEP:<br>W1-W2= −.00;<br>W2-W3= .00<br>p>0.05<br>SMS → SLEEP:<br>W1-W2= .05;<br>W2-W3= .05<br>p>0.05<br><br>*Cross-lagged panel model*<br>**Daytime sleepiness**<br>**Between-person level:**<br>SM use b*=0.35, p<0.001<br>SM stress b*=0.61, p<0.001<br><br>**Within person level:**<br>SMU → SLEEP:<br>W1-W2= −.05<br>W2-W3= −.04	between the random intercept factors of SM use and sleep latency, while relationship between SM stress and sleep latency were significant. This implies adolescents who reported higher levels of SM stress reported longer sleep latency across the three waves.<br><br>Within person level – SM use and SM stress did not further increase sleep latency among adolescents over time.<br><br>For daytime sleepiness, the between person

			5 items indicating SM dependency: have you (1) neglected other activities to use social media; (2) had the need to use social media more often or for a longer time; (3) spent little time with friends/family because you were using social media; (4) frequently checked social media to see if you had received a new message, and (5) unsuccessfully tried to limit your social media use.		$p>0.05$<br>SMS → SLEEP:<br>W1-W2= .04<br>W2-W3= .05<br>$p>0.05$	correlations showed adolescents who more frequently engaged in SM use and reported higher SM stress showed more daytime sleepiness.<br><br>At the within-person level, SM use and SM stress did not further increase daytime sleepiness among adolescents.
Maksniemi et al., 2022	Finland<br><br>Longitudinal<br><br>Follow-up: 6 years (5 collection timepoints between 2014-2019)	N=426<br><br>Age: started at 13-14 years and finished at 18-19 years<br><br>Sex: 65% female	***Active social media use***<br>The intensity of social media use and level of engagement measured using the Social-media-networking dimension of the Socio-Digital Participation Inventory. Four items<br>(1) "I chat"; (2) "I visit and send messages vis social media sites"; "I post updates or share	***Bedtime***<br>Self-reported bedtime was assessed with one question: "What time do you usually go to bed when you have school the next morning."	*Random intercept cross-lagged panel model (controlled for participants' sex)*<br><br>Model-based standardized within- and between-level correlations linking active social media use and bedtime	There was no clear pattern between active social media use, and bedtime across adolescence – statistically significant associations varied depending on the measurement time.

			interesting content"; and (4) "I post pictures or picture updates" on a 7-point frequency scale (1=never; 2=a couple of times a year; 3=monthly; 4=weekly; 5=daily; 6=multiple times a day; 7=all the time).		Time 1:<br>r = 0.143, 95% CI 0.003 to 0.283, p=0.045<br><br>Time 2 (correlated change):<br>r=0.036, 95% CI -0.135 to 0.207, p=0.679<br><br>Time 3 (correlated change):<br>r= -0.156, 95% CI -0.373 to 0.061, p=0.158<br><br>Time 4 (correlated change):<br>r= 0.041, 95% CI -0.153 to 0.236, p=0.677<br><br>Time 5 (correlated change):<br>r= 0.060, 95% CI -0.098 to 0.219, p=0.456	Active social media use was associated with delayed bedtimes only in early adolescence when participants were aged 13-14.
**IED screen time/brightness**						
Patte, 2017	Canada	N=26,205<br><br>Age: grade 9-12	***Screen time:*** A survey which asked participants the average time per day	***Sleep duration:*** Assessed by asking how much time in hours (0–9) and minutes (0, 15, 30, 45)	*Logistic regression (adjusted for*	No longitudinal effect was observed when

	Longitudinal cohort study<br><br>Follow-up: 4 years	Baseline mean age: not reported<br><br>Sex: 55% female<br><br>Ethnicity: 71% Caucasian, 2.5% Black	that they spent using a screen: "talking on the phone," "surfing the internet," "texting, messaging, emailing," and "doing homework".	participants usually spend sleeping per day. Responses were classified as either "meets recommendations" (≥8 h) or "insufficient sleep" (< 8 h).	*(gender, grade, race/ethnicity)*<br><br>**Talking on the telephone:** OR= 1.01 (95%CI 0.98 to 1.03)<br><br>**Surfing the internet:** OR = 1.01 (95% CI 1.00 to 1.02)<br><br>**Texting, messaging, or emailing:** OR= 1.00 (95%CI 0.99 to 1.01	students increased their screen use of any type.<br><br>Based on the cross-sectional effects, youth spending more time surfing the internet or playing video/computer games had a greater risk of inadequate sleep.
Perrault et al., 2019	Switzerland<br><br>Intervention (non-RCT)<br><br>Duration of intervention: 2 weeks	N=569<br><br>Age: 12-19<br>Baseline mean age: 15.35 SD 2.1<br><br>Sex: 52% female	***Screen time (after 9pm from Sunday to Thursday evenings):***<br><br>After 2 weeks baseline (phase 1) data collection (no instruction to change screen use), participants were instructed to stop using screen devices after 9pm on school evenings (Sunday to Thursday) for 2 weeks.<br><br>Brainstorming conducted to come up with off-	***Light off time, sleep onset time, wake up time, out of bed time, time in bed, total sleep period, total sleep time, sleep efficiency:*** Measured using a combination of diaries and actigraphy.<br><br>Diaries: Participants reported light off time, time to fall asleep (i.e. sleep latency), wake-up time (i.e. time of morning awakening), out of bed time, and frequency of nocturnal awakenings. Participants also evaluated their sleep quality and morning mood	*ANOVA/t-test*<br>**Light off time (HH:MM):**<br>Follow-up: 22:48 (SE=3mins) p<0.001<br>**Wake-up time (HH:MM):**<br>Follow-up: 06:58 (SE=2mins) p=0.41<br>**Out-of-bed time (HH:MM):**<br>Follow-up: 07:10 (SE=2mins) p=0.24	Decreased screen time in evening was associated with advanced light off time, sleep onset time and increased sleep duration, especially in older adolescents.

			screen activities to engage in after 9pm (eg. involve family, play music, read books).	using a 5-star rating system (1: very bad sleep to 5: very good sleep, and 1: very bad mood to 5: very good mood).<br><br>Actigraphy: wore an Actimeter GT3X+ (Actigraph, Pensacola, FL) non-stop for 2 successive periods of 2 weeks. Mean actigraphic data during 60s epochs were scored as sleep or wake using an automatic detection algorithm. Reviewed each night manually, by comparing the sleep onset times indicated by the analyses of the actimetry data with those reported by participants in diaries.<br>Data from any night where a 1 hr mismatch between subjective and objective wake-up times was observed were excluded (6.4% excluded).<br><br>The following sleep variables were obtained during school nights and weekend nights:<br>Light off time (diary),<br>sleep onset time (actigraphy and diary),<br>wake-up time (actigraphy and diary); out-of-bed time (diary),	**TiB (HH:MM):**<br>Follow-up: 08:21 (SE=3mins)<br>$p<0.001$<br>**TSP (HH:MM):**<br>Follow-up: 07:51 (SE=3mins)<br>$p<0.001$<br>**Main effect phase:**<br>$F(1,179)=44.03$<br>$p<0.001$<br>**Main effect age:**<br>$F(3,179) = 11.14$<br>$p<0.001$<br>**Interaction phase x age:**<br>$F(3,179) = 5.23$<br>$p=0.002$<br>**TST (HH:MM):**<br>Follow-up: 07:23 (SE=3mins)<br>$p<0.001$<br>**SE (%):**<br>Follow-up: 88.49 (SE=0.35)<br>$p=0.24$<br><br>ANOVA<br>**Main effect phase:**<br>$F(1,444)=56.42$<br>$p<0.001$	

				time in bed (TiB; period between light_off time and out-of-bed time), total sleep period (TSP; period between sleep onset time and wake-up time), total sleep time (TST; TSP minus wake period after sleep onset time), sleep efficiency (SE in %: TST/TiB*100).<br><br>**Chronic Sleep Reduction:** Chronic sleep reduction questionnaire (CSRQ).	**Main effect participant group:**<br>F (1,443) = 0.91<br>p=0.34<br>**Interaction phase x participant group:**<br>F (1,1) =4.86<br>p=0.028	
Yoo, 2020b	Korea<br><br>Comparative analysis between 2 longitudinal cohorts, separated by 3 years<br><br>Follow-up: 4 years<br><br>Sex: 48.5% female	N=2081 (2000 birth cohort)<br>N=2254 (1997 birth cohort)<br><br>Age: grade 7-10<br>Mean age: 2000 birth cohort 12.95-15.95; 1997 birth cohort<br>12.90 - 15.90	***Playing computer games:*** Time spent playing games measured with one parameter:<br>“How much time do you spend playing games during the day?”	***Sleep duration (SD):*** Specific question asked "Exactly what time (hour and minutes) do you sleep and what time (hour and minutes) do you wake up?”. SD calculated as difference between sleep time and waking time.	*Latent growth curve modelling (LGCM) and multi-group analysis (MGA)*<br><br>**2000 birth cohort**<br>Sleep duration intercept:<br>B = 0.021<br>β = 0.042<br>SE = 0.026<br>p>0.05<br>Sleep duration slope:<br>B = -0.086<br>β = -0.272<br>SE = 0.037<br>p<0.05<br>Sleep duration quadratic:<br>B = 0.031	In the 2000 birth cohort, the more time spent on playing games, showed the slower the sleep duration linear increase, and the slower sleep duration quadratic decrease.<br><br>In the 1997 birth cohort, no significant relationship between playing games and sleep duration.

					β = 0.356<br>SE = 0.012<br>p<0.05<br><br>**1997 birth cohort**<br>Sleep duration intercept:<br>B = -0.054<br>β = -0.065<br>SE = 0.028<br>p>0.05<br>Sleep duration slope:<br>B = 0.028<br>β = 0.051<br>SE = 0.030<br>p>0.05<br>Sleep duration quadratic:<br>B = -0.005<br>β = -0.035<br>SE = 0.010<br>p>0.05<br><br>Critical ratio for difference (CRD, 1997 vs 2000)<br>Sleep duration slope:<br>2.409, p<0.05<br>Sleep duration quadratic:<br>-2.253, p<0.05	Time spent on playing games had a greater impact on sleep duration in the more recent birth cohort.

Heath et al., 2014	Australia<br><br>Within-subject controlled study	N=16<br><br>Age: 14-19<br>Mean age: 17.4 (sd1.9)<br><br>Sex: 56% female	***Apple iPad 2 used as light-emitting technological device, with three counterbalanced conditions: bright unfiltered screen light (80 lux), f.lux short-wavelength filtered light (50 lux), dim light (1 lux).***<br><br>Participants attended sleep lab for 3 nights over 3 weeks - preferably same school night each week.<br><br>17:00 – Arrival, followed by quiet activities.<br>18:30 - Dinner<br>19:00 - All technological devices taken away.<br>Two hours before bedtime (according to participant usual bedtimes) participants completed 1 hour of dark habituation (quiet activities in a dimly lit room <10 lux) (room temp 22°C).	**Subjective sleepiness:**<br>Stanford Sleepiness Scale<br>**Sleep onset latency and slow rolling eye movements (SREMs):**<br>Polysomnography, EEG, EOG and EMG measurements taken using a portable Compumedics Somte.<br><br>A trained sleep technician (blind to conditions) calculated sleep onset latency as the time between ''lights out'' and the first of three 30-sec epochs of stage 1 or 2 sleep, whether slow-rolling eye movements (SREM) were present or not in each 30-sec epoch during the sleep onset process, and slow-wave sleep (SWS) and REM sleep as per Rechtschaffen and Kales (1968) scoring criteria.	*Mixed-model ANOVA*<br><br>**Main effect screenlight:**<br>$F(2, 30)= 1.71$<br>$\eta^2= 0.10$<br>$p=0.2$<br><br>**Time effect:**<br>$F(1.37, 20.52)= 23.21$<br>$\eta^2= 0.61$<br>$p<0.001$<br><br>**Interaction light x time:**<br>$F(4, 60)= 2.34$<br>$\eta^2= 0.14$<br>$p=0.07$<br><br>*Repeated measures ANOVA*<br><br>**Screenlight effect on SOL:**<br>$F(2, 22)= 0.00$<br>$\eta^2= 0.00$<br>$p=0.99$	No significant difference was observed between bright, dim, and filtered short-wavelength (f.lux) screen light in subjective assessments of self-rated pre-sleep sleepiness.<br><br>No significant differences were observed for subjective and objective SOL, SREMs, minutes of SWS and REM, or morning functioning between bright, dim, and filtered short-wavelength (f.lux) screen light.

			In both bright and f.lux conditions, screen brightness was set to the highest setting and then to the lowest setting for the dim condition. iPad exposure in all three conditions was performed in darkness. Adolescents sat in semi-upright position while holding the iPad 40cm away from the face.<br><br>On each testing night iPad exposure totalled 48 min, split into two 24-min segments.<br>First 16 min of each segment, adolescents' played 1 of 6 games, and then watched a video compilation for following 8 min.<br><br>Videos and games were randomized across conditions. Selected games and videos all featured a white background, to reduce lux variation and produce maximal alertness.		**Screenlight effect on SREMS:**<br>$F(2, 22)= 0.98$<br>$\eta^2= 0.08$<br>$p=0.39$<br><br>**Minutes of SWS and REM sleep:**<br>1st NREM-REM cycle<br>$F(2,22)= 0.21$<br>$\eta^2 = 0.02$<br>$p= 0.81$<br><br>2nd NREM-REM cycle<br>$F(2,22)= 1.37$<br>$\eta^2 = 0.11$<br>$p= 0.28$<br><br>**Screenlight effect on morning functioning:**<br>$F(2,30)= 0.09$<br>$\eta^2 = 0.92$<br>$p= 0.01$	

			In conjunction with three 4-min GO/NOGO tasks, adolescents experienced 1 hr of screen light exposure.<br><br>After completion of final sleepiness scale adolescents were allowed to sleep.			
**Adverse implications of IED use**						
IED overuse or problematic use						
Lee et al., 2017	Korea<br><br>Longitudinal<br><br>Wave 1 = 2011<br>Wave 2 = 2012<br>Wave 3 = 2013	N=<br>Wave 1 = 1,644<br>Wave 2 = 1,125<br>Wave 3 = 638<br><br>Age:<br>Wave 1 = 15<br>Wave 2 = 16<br>Wave 3 = 17<br><br>Sex:<br>49% female	***Mobile phone addiction:*** Tool developed through discussion with a specialist and systematic review: "The amount of time using my cell phone is increasing", "I feel nervous without my cell phone", "I feel nervous when I have not received any message or call in some time", "I'm easily unaware of the passing of time when I'm using my cell phone", "I feel isolated when I don't have my cell phone with me", and "I feel too uncomfortable to live even a day when I don't	***Sleep quality:*** Subjects asked to rate how well they sleep; poor quality sleep defined as follows: "You cannot fall asleep deeply, and wake up often during the night". 4 possible responses: very well, well, poorly, or very poorly. "Poorly" or "very poorly," indicated poor sleep quality.<br><br>***Sleep duration:*** Subjects asked "What time did you go to sleep and get up on average on weekdays (Monday to Friday) this semester?"	**Mobile phone addiction score (OR, 95% CI):**<br>Low (≤ 15)<br>Poor sleep quality = 1.000; Sleep Duration = 1.000<br>Middle (> 15 and ≤ 20)<br>Poor sleep quality = 1.313 (0.991–1.740); Sleep Duration= 1.030 (0.819–1.295)<br><br>High (> 20)<br>Poor sleep quality = 2.009 (1.443–2.796); Sleep	Increased mobile phone addiction was associated with a higher risk of poor sleep quality, but had no significant effect on sleep duration.<br><br>Gender differences were identified in the relationship between mobile phone addiction and sleep quality.

			have my cell phone with me".<br><br>Mobile phone addiction level was categorized into 3 groups: low (mobile phone addiction score ≤ 15), middle (mobile phone addiction score > 15 and ≤ 20), and high (mobile phone addiction score > 20)		Duration= 1.021 (0.767–1.360)<br><br>**Male (OR, 95% CI)**<br>Middle = 1.448 (0.994-2.108)<br>High = 1.726 (1.061-2.806)<br>**Female**<br>Middle= 1.175 (0.764-1.807)<br>High= 2.221 (1.392-3.543)	
Yoo, 2020a	Korea<br><br>Longitudinal<br><br>Follow-up duration: 6 years	N=2257<br><br>Age: 11.9-16.98<br>Age mean: Wave 1 (11.90 sd 0.34)<br>Wave 2 (12.89 sd 0.34)<br>Wave 3 13.89 sd 0.34)<br>Wave 4 (14.89 sd 0.34)<br>Wave 5 (15.89 sd 0.35)<br>Wave 6 (16.89 sd 0.35)<br><br>Sex: 49.6% female	***Mobile phone dependency:*** Seven items: 1. My mobile phone usage time is increasing more and more; 2. I'm nervous if I do not have my mobile phone; 3. I'm nervous if no one calls my mobile phone for a long time; 4. I do not know how much time I spend on my mobile phone; 5. when I'm alone without my mobile phone I am so bored that I cannot stand it; 6. if I do not have a mobile phone, I feel isolated; 7. I cannot live without my mobile phone	***Sleep duration:*** Specific question asked "Exactly what time (hour and minutes) do you sleep and what time (hour and minutes) do you wake up?"; sleep duration calculated as difference between sleep and waking times.	*Hierarchical linear modelling (HLM)*<br><br>**Unconditional model:**<br><br>µintercept = 7.899<br>SE (0.021)<br>p<0.001<br>µslope = 0.393<br>SE (0.007)<br>p<0.001<br>βτ01 = -0.477<br>p<0.001	Mobile phone dependency was not found to be significantly related to the decreasing sleep duration trajectory over 6 years

		Attrition rate 3%, 3.9%, 10.3%, 11.1%, 12.5%, and 20% for each wave respectively.	because I am uncomfortable without it.<br><br>All items scored on 4-point Likert scale; higher score indicates a higher level of mobile phone dependency.			
Yoo, 2020b	Korea<br><br>Comparative analysis between 2 longitudinal cohorts, separated by 3 years<br><br>Follow-up: 4 years<br><br>Sex: 48.5% female	N=2081 (2000 birth cohort)<br>N=2254 (1997 birth cohort)<br><br>Age: grade 7-10<br>Mean age: 2000 birth cohort 12.95-15.95; 1997 birth cohort 12.90 - 15.90	***Smartphone overuse:*** Measured using seven items, such as “My mobile phone usage time is increasing more and more.”<br><br>All items were answered using a four-point Likert scale. A higher score indicates a higher level of smartphone overuse.	***Sleep duration (SD):*** Specific question asked "Exactly what time (hour and minutes) do you sleep and what time (hour and minutes) do you wake up?”. SD calculated as difference between sleep time and waking time.	*Latent growth curve modelling (LGCM) and multi-group analysis (MGA)*<br><br>**2000 birth cohort**<br>SD intercept:<br>B = -0.014<br>β = -0.118<br>SE = 0.004<br>p<0.01<br>SD slope:<br>B = 0.012<br>β = 0.156<br>SE = 0.006<br>p<0.05<br>SD quadratic:<br>B = -0.004<br>β = -0.179<br>SE = 0.002<br><br>**1997 birth cohort**<br>SD intercept:<br>B = 0.003<br>β = 0.017	In the 2000 birth cohort, greater smartphone overuse yielded a shorter sleep duration linear increase, however there was no related quadratic change rate.<br><br>In the 1997 birth cohort, there was no significant relationship between smartphone overuse and sleep duration.<br><br>Smartphone overuse had a greater impact on sleep duration in recent

					SE = 0.004<br>SD slope:<br>B = -0.007<br>β = -0.058<br>SE = 0.005<br>SD quadratic:<br>B = 0.002<br>β = 0.070<br>SE = 0.002<br><br>CRD intercept:<br>2.800<br>p<0.01<br>CRD slope:<br>-2.542<br>p<0.05<br>CRD quadratic:<br>2.353<br>p<0.05	generations than in past generations.
Kojima et al., 2019	Japan<br><br>Repeated Cross-sectional study<br>Each year between 2014-2016	N=2887<br><br>Age: 12-15 years<br><br>Boys = 49.8%	***Problematic Internet use (PIU):*** Internet Addiction Test (IAT) used to assess PIU.<br><br>IAT consisted on 20 questions related to internet use. Internet use included online gaming, social networking (e.g., LINE and Twitter), video downloading (e.g., YouTube), personal website browsing or	***Lifestyle habits:*** Assessed by questions related to degree of sleepiness after awakening in the morning, skipping breakfast, exercise habits, weekday study time, and bedtime.<br><br>Questions based on a previous report conducted by the Ministry of Education, Culture, Sports, Science and Technology in Japan.<br><br>Questions scored on 5-point scale. Scores ranged from 1 to 5,	*Logistic regression analysis (adjusted by sex, person of trust to talk to, breakfast, exercise habits, studying time, bedtime, depression, and orthostatic dysregulation).*<br>**Bedtime OR(95% CI):**<br>On or before 22.59 hrs= ref	A significant positive association was identified between PIU and having a late bedtime (after 24.00 hrs) among students from all grades.<br><br>A significant positive association was

			blogging, and online shopping.	and total score ranged from 20-100 points.<br><br>Scores ≥70 points classified as 'severe PIU'; 69–40 classified as 'mild PIU'; and ≤39 classified as 'normal'.	23.00-23.59 hrs= 1.56 (0.94 — 2.59)<br>After 24.00 hrs= 2.29 (1.37 — 3.81)<br><br>**Sleepiness after wakening in the morning OR(95% CI):**<br>Sleepy= 3.45 (1.67 — 7.12)<br>A little sleepy= 2.03 (1.01 — 4.07)<br>Well= ref	identified between PIU and sleepiness after awakening in the morning, except among 1st grade junior high school students.
Chang et al., 2022	Taiwan<br><br>Longitudinal cohort study<br><br>Follow-up: 1 year (5th to 6th grade)	N=2155<br><br>Age not reported. Taiwan grade 5 and 6 (ages 10 and 11 respectively)<br><br>Sex: 47.4% female<br><br>25.8% reported that their household income was of the lower or lower-middle class.	***Smartphone addiction***<br><br>Assessed using the short-form Smartphone Addiction Inventory: 10 items to assess symptoms of smartphone addiction, each item was evaluated on a 4-point Likert type scale that ranged from "strongly agree" (scoring 4) to "strongly disagree" (scoring 1). The total score of the scale ranged from 10 to 40. Children with a score of 25 or higher were classified as having a smartphone addiction.	***Sleep problems***<br>Categorised into two outcomes:<br>**(1) Inadequate sleep quantity**<br>**(2) Poor sleep quality**<br><br>Questions were adapted from the Chinese version of the Pittsburgh Sleep Quality Index.<br><br>Inadequate sleep quantity assessed by asking participants to list their sleep patterns during the past month and characterize whether total sleep time was (a) sufficient (at least 3 days/week), (b) somewhat, (c) moderately, or (d) seriously insufficient. Participants who answered (b), (c)	*Multiple logistic regression (sex, academic performance, parental marital status & household income included in model)*<br>*Two groups identified and compared to reference group (with no problems with sleep quality, sleep quantity or depression):*<br>*Onset group – those who reported no problems during the*	The onset of inadequate sleep quantity was predictable for children who reported an increase in smartphone addiction from grades 5 to 6<br><br>The persistence of inadequate sleep quantity was predictable for children who reported an increase in smartphone

				or (d) were categorised as having inadequate sleep quantity.<br><br>Poor sleep quality was assessed by asking participants to list their sleep patterns during the past month and assess their feelings about overall sleep quality as (a) very satisfied, (b) not good, (c) obviously bad, or (d) very unsatisfactory. If they answered (b), (c) or (d) they were categorised as having poor sleep quality.	*5th grade but did report problems in the 6th grade*<br>*Persistence group – those who reported problems in both the 5th and 6th grades.*<br><br>**Predictors of the onset of inadequate sleep quantity:** Smartphone addiction (6th-5th grade) OR 2.23 (95% CI 1.57 to 3.15)<br><br>**Predictors of the persistence of inadequate sleep quantity:** Smartphone addiction (6th-5th grade) OR 1.96 (95% CI 1.41 to 2.73)<br><br>**Predictors of the onset of poor sleep quality:**	addiction from grades 5 to 6.<br><br>The onset of poor sleep quality was predictable for children who reported an increase in smartphone addiction from grades 5 to 6<br><br>The persistence of poor sleep quality was predictable for children who reported an increase in smartphone addiction from grades 5 to 6.

					Smartphone addiction (6th-5th grade) OR 1.92 (95% CI 1.36 to 2.72)<br><br>**Predictors of the persistence of poor sleep quality:** Smartphone addiction (6th-5th grade) OR 2.14 (95% CI 1.52 to 3.04)	
Telepressure						
Barber & Santuzzi, 2017	USA<br><br>Longitudinal cohort study<br><br>Follow-up: 5-9 weeks	N=241<br><br>Age: 18-28 years Baseline mean age: 19.0 (SD 1.8)<br><br>Sex: 58% female<br><br>Ethnicity: 64.0% White/European, 15.6% Black/African-American, 10.5% Latino/Hispanic, 4.6% Asian, 4.0% Biracial/Multi-racial.	***Telepressure:*** Assessed using a 6 item scale, which asked participants to rate the extent to which they agree (1=strongly disagree; 5 = strongly agree) with statements on social interaction using information-communication technology (e.g. phones, emails). Items included:<br><br>1. It's hard for me to focus on other things when I receive a message for someone.	***Sleep hygiene***: Measured using 13-item Sleep Hygiene Index.<br><br>Participants asked to respond to statements, such as "I go to bed at different times from day to day". Response options ranged from 1 (never) to 5 (always). Higher total score = poorer sleep hygiene.	*Bivariate correlation:* r=0.18, p<0.05<br><br>*Multiple regression:* b= 0.11 (SE=0.04), p<0.05<br><br>No adjustment for confounders.	A correlation was observed between telepressure at the beginning of the semester and poor sleep hygiene 1 month later.<br><br>An increase in telepressure was associated with poorer sleep hygiene.

		Employment status:<br>Part 1<br>59.1% not employed<br>36.1% part-time<br>4.8% full-time<br>Part 2<br>87% no change in status<br>5.1% newly employed<br>7.9% lost employment<br><br>Other: All participants recruited from an Introductory Psychology course at a 4-year public university.	2. I can concentrate better on the tasks once I've responded to my messages.<br>3. I can't stop thinking about a message until I've responded.<br>4. I feel a strong need to respond to others immediately.<br>5. I have an overwhelming feeling to respond right at that moment when I receive a request.<br>6. It's difficult for me to resist responding to a message right away.			
Cybervictimisation						
Jose & Vierling, 2018	New Zealand<br><br>Longitudinal cohort study<br><br>Follow-up: 1 year & 2 years	N=2179 (baseline)<br><br>Age: 10-15 years<br>Baseline mean age: not reported	***Cybervictimisation:*** defined as being a victim of cyber-aggression (persistent, hurtful acts perpetrated on another individual through electronic text or pictures).	***Sleep adequacy:*** Measured using one survey question, “In the last week, on how many nights did you get at least 8 hrs of sleep?”.<br><br>Responses provided on a scale of 0 to 7 days.	*Bivariate correlation:*<br><br>1 year: $r= -0.09$, $p<0.01$	Cybervictimisation predicts lower levels of sleep one year later.

		Sex: 52% female<br><br>Ethnicity: 59% New Zealand European, 28% Māori, and 15% other	Assessed asking two questions:<br>1. "In the last month, about how often have you received a mean text message from someone?"<br>2. "In the last month, how often have you been bullied by others online?"<br>Two items averaged to produce a single score. Responses ranged from 1 ("never"), 2 ("1 to 3 times"), 3 ("4 to 6 times"), 4 ("7 or more times") to 5 ("almost daily/daily").		2 years: r= -0.04, p= >0.05 (value not reported)<br><br>*Regression model (adjusted for sex, age, ethnic group):*<br><br>1 year: β=−0.05, p=0.008<br><br>Averaged over 2 years: β=−0.08, p=0.011	
Patte, 2017	Canada<br><br>Longitudinal cohort study<br><br>Follow-up: 4 years	N= 26,205<br><br>Age: grade 9-12 Baseline mean age: not reported<br><br>Sex: 55% female<br><br>Ethnicity: 71% Caucasian, 2.5% Black	***Cybervictimisation:*** assessed using a single question "In the last 30 days, in what ways were you bullied by other students?" Response option: cyber-attacks (e.g., being sent mean text messages or having rumours spread about you on the internet) Response options included: "I have not	***Sleep duration:*** Assessed by asking how much time in hours (0–9) and minutes (0, 15, 30, and 45) participants usually spend sleeping per day. Responses were classified as either "meets recommendations" (≥8 h) or "insufficient sleep" (< 8 h)	Logistic regression (adjusted for (gender, grade, race/ethnicity)<br><br>Adjusted OR=0.82 (95%CI 0.74 to 0.91)	Relative to baseline, students became less likely to meet the sleep recommendations if, at follow-up, they had experienced cybervictimisation

			been bullied in the last 30 days:" YES/NO scale			
Herge et al., 2016	USA<br><br>Repeated cross-sectional<br>3 time points, 6 weeks apart	N= 1,162<br><br>Age: 13–19 years<br>Mean age: 15.80<br>SD=1.2<br><br>Sex: 57% female<br><br>Ethnicity: 80% Hispanic;<br>Race:<br>84% White, 12% Black, 4% Asian	***Cyber peer victimisation (PV):*** At Time 1, a Cyber Peer Experiences Questionnaire assessed cyber PV using nine items. Items included, "a peer posted pictures of me that made me look bad via electronic media", "a peer sent me a mean message via electronic media", "a peer posted mean things about me publicly via electronic media".	***Sleep problems:*** Three items assessed sleep problems at Time 3, of which two sleep deficit items ("I have trouble falling asleep," and "I have trouble staying asleep") were derived from PTSD-Reaction Index (Steinberg, Brymer, Decker, & Pynoos, 2004).<br>One sleep excess item ("I sleep more than usual") was derived from depression measures appropriate for youth, such as the Beck Depression Inventory (Beck, Steer, & Brown, 1996).	*Structural equation modelling*<br><br>Time 1 cyber peer victimisation and sleep symptoms (Excess sleep and Sleep deficit) at time 3<br><br>Excess sleep β = 0.14, p<0.05<br>Sleep deficit β = 0.17, p<0.05	Negative peer experiences, that occur via technology, may contribute directly to adolescents' sleep difficulties.
Chang et al., 2022	Taiwan<br><br>Longitudinal cohort study<br><br>Follow-up: 1 year (5th to 6th grade)	N=2155<br><br>Age not reported.<br>Taiwan grade 5 and 6 (ages 10 and 11 respectively)<br><br>Sex: 47.4% female	***Online harassment***<br>Measured using 3 questions adapted from the US Youth Internet Safety Surveys.<br>Participants asked: during the past year did someone (1) send you sexual content that you did not want; (2) ask you to talk about sex online when you did not want to; (3) ask you to do	***Sleep problems***<br>Categorised into two outcomes:<br>**(1) Inadequate sleep quantity**<br>**(2) Poor sleep quality**<br><br>Questions were adapted from the Chinese version of the Pittsburgh Sleep Quality Index.<br><br>Inadequate sleep quantity assessed by asking participants to list their sleep patterns during the	*Multiple logistic regression (sex, academic performance, parental marital status & household income included in model)*<br>*Two groups identified and compared to reference group (with no problems*	The onset of inadequate sleep quantity was predictable for children who reported an increase in online harassment from grades 5 to 6<br><br>The persistence of inadequate sleep quantity was

		25.8% reported that their household income was of the lower or lower-middle class.	something sexual online that you did not want to do? If a participant answered “yes” for any of the three items they were categorised as having experienced online harassment.	past month and characterize whether total sleep time was (a) sufficient (at least 3 days/week), (b) somewhat, (c) moderately, or (d) seriously insufficient. Participants who answered (b), (c) or (d) were categorised as having inadequate sleep quantity.<br><br>Poor sleep quality was assessed by asking participants to list their sleep patterns during the past month and assess their feelings about overall sleep quality as (a) very satisfied, (b) not good, (c) obviously bad, or (d) very unsatisfactory. If they answered (b), (c) or (d) they were categorised as having poor sleep quality.	*with sleep quality, sleep quantity or depression):*<br>*Onset group – those who reported no problems during the 5th grade but did report problems in the 6th grade*<br>*Persistence group – those who reported problems in both the 5th and 6th grades.*<br><br>**Predictors of the onset of inadequate sleep quantity:**<br>Online harassment (6th-5th grade) OR 1.76 (95% CI 1.01 to 3.05)<br><br>**Predictors of the persistence of inadequate sleep quantity:**<br>Online harassment (6th-5th grade) OR 2.06 (95% CI 1.25 to 3.42)	predictable for children who reported an increase in online harassment from grades 5 to 6.<br><br>The onset of poor sleep quality was predictable for children who reported an increase in online harassment from grades 5 to 6<br><br>The persistence of poor sleep quality was predictable for children who reported an increase in online harassment from grades 5 to 6.

					**Predictors of the onset of poor sleep quality:**<br>Online harassment (6th-5th grade) OR 1.91 (95% CI 1.13 to 3.24)<br><br>**Predictors of the persistence of poor sleep quality:**<br>Online harassment (6th-5th grade) OR 1.60 (95% CI 0.91 to 2.81)	
**Positive implications of IED use**						
Smartphone applications as sleep aids						
Werner-Seidler et al., 2019	Australia<br><br>Pilot study (single arm pre-post intervention design)<br><br>Follow-up: 6 weeks	N=50 (baseline)<br><br>Age: 12 to 16 years<br>Baseline mean age: 13.71 (SD 1.35)<br><br>Sex: 66% female<br><br>Other: All experienced mild insomnia; 94% born in Australia	***Sleep Ninja App:*** App aimed to teach users about the importance of consistent sleep and wake times, and recommended bedtimes.<br><br>The app included six training lessons, a sleep tracking function, recommended bedtimes based on sleep guidelines, reminders to start wind-down routine each night, sleep tips and	***Insomnia***: Measured using Insomnia Severity Index; higher scores = more severe insomnia.<br><br>***Sleep Quality***: Measured using Pittsburgh Sleep Quality Index; higher scores = poorer quality.<br><br>***Sleep Onset Latency***: Defined as time taken to fall asleep.	β=−4.29 (95%CI −5.63 to 2.95)<br><br>β=−1.88 (95%CI −2.85 to 0.90)<br><br>ß= −0.37 (95%CI −0.70 to −0.03)	Insomnia symptoms improved significantly from baseline to post-study.<br>An improvement in self-reported sleep quality was observed post-study.<br>A significant decrease (21 mins) in time taken to fall asleep was

			general sleep information.<br>Users received a prompt 1-hour before bedtime to commence prebed time routine and encouraged to stop using electronic devices.<br><br>Training sessions, delivered through a chat-bot format, took 5–10 minutes to complete.<br><br>Development informed by cognitive behavioural therapy for insomnia.	***Night-time Awakenings (NWAK):*** Defined as number and duration of night time awakenings.<br><br>***Sleep Refreshingness***: Measured on scale from 1 = exhausted to 5 = very refreshed.<br><br>***Use of Sleep Medication***: Measured as proportion of days taking medication.<br><br>***Total Sleep Time:*** Calculated by subtracting sleep-onset latency, wake after sleep onset (WASO), and time between waking and getting up in the morning, from time in bed.<br>***Time in Bed (TIB):*** Defined as total time spent in bed (awake and sleeping)**.**<br><br>***TIB after Final Morning Wake Up:*** Defined as time between waking and getting out of bed.	ß=−0.46 (95%CI −0.81 to −0.11)<br><br>ß=0.43 (95%CI 0.19 to 0.68)<br><br>ß=−0.01 (95% CI −0.02 to 0.01)<br><br>ß=0.53 (95%CI 0.17 to 0.90)<br><br>ß=−0.01 (95%CI −0.42 to 0.41)<br><br>ß=−0.27 (95% CI −0.41 to −0.12)	observed post-study.<br>A significant reduction was observed post-study in number of times participants woke during night.<br>An improvement was observed in how refreshing participants reported their sleep to be post-study.<br>No significant difference post-study.<br><br>An improvement, of 31 minutes, was observed in TST post-study.<br><br>No significant difference post-study.<br><br>Participants spent significantly less

				Habitual sleep efficiency: Measured TST as a percentage of TIB (TST/TIB).	ß=5.25 (95%CI 1.03 to 9.47)	TIB after waking post-study.<br><br>An improvement in sleep efficiency was observed post-study.

Supplementary Table 4. Summary of findings on the relationship between IED use and mental health, mediated by the impact of IED use on sleep

Reference	Study characteristics	Sample characteristics	Exposure/Intervention Description	Sleep mediator description	Outcome description	Findings	Narrative Findings
**Interactive electronic device (IED) use**							
Vernon, 2018	Australia<br><br>Longitudinal cohort study<br><br>Follow-up: 1 year	N=1101<br><br>Age: 13-16 years Baseline mean age: 13.5 years<br><br>Sex: 57% female<br><br>Ethnicity: 56.9% Caucasian, 7.1% Asian, 2% Aboriginal or Torres Strait Islander, 21.9% other<br><br>Other: 44% from lower socio-economic status	***Night-Time Mobile Phone Use:*** Students were asked if they had a mobile phone. If answered yes, they were asked, "At what time of the night do you usually send or receive messages and/or phone calls?"<br><br>6 response options included: never text or phone after lights out; immediately after lights out; 10–11 p.m.; 11 p.m.–12 a.m.; 12–1 a.m.; 1–2 a.m.; 2–6 a.m.; at any time of the night.<br><br>Coded on 6-point scale (0-5) as 0 = no mobile phone, 1 = never text or phone after lights out, 2 = immediately after lights out, 3 = before midnight, 4 = after midnight, and 5 = at any time of the night	***Sleep quality:*** Assessed using a scale consisting of the mean of eight items drawn from School Sleep Habits Survey. The sleep scale tapped perceptions about sleep quality and behaviour during the previous 2 weeks.<br><br>Responses were 1 = never, 2 = once, 3 = twice, 4 = several times, and 5 = every day/night. Higher scores = lower sleep quality	***Depressed mood:*** Questionnaire from Michigan Study of Adolescent Life Transitions, comprising mean of 5 items.<br><br>Items included: "How often do you feel there is nothing nice you can look forward to; feel unhappy, sad, or depressed?"<br><br>Responses ranged from 1 (never) to 6 (daily). Higher scores = worse outcome	*Latent Growth Curve Mediation Models (Mediator: sleep quality; Covariate: bedtime, gender, SES)*<br><br>**Depressed mood: Indirect effect slopes:** *B*=0.29 (95%CI 0.10 to 1.75), β=0.44<br><br>***Externalising behaviour:*** **Indirect effect slopes:** *B*=0.08 (95%CI 0.01 to 1.34), β=0.12<br><br>**Self-esteem:**	Both night-time mobile phone use and poor sleep behaviour underwent positive linear growth over time.<br><br>Changes in sleep behaviour mediated the relation between early changes in night-time mobile phone use and later increases in depressed mood, externalising behaviour, self-esteem and coping.

					Externalising behaviour: Questionnaire comprising mean of 7 items.<br>Items included: "In the past 6 months how often have you skipped school without parent permission?; how often have you gotten in a physical fight with another person?"<br><br>Items measured on 8-point scale from 1 (none) to 8 (31 or more times), Higher scores = worse outcome.<br><br>**Self-esteem:** Questionnaire, comprising mean of 3 items.	**Indirect effect slopes:** *B*=0.13 (95%CI 0.04 to 0.73), β=0.15<br><br>***Coping:*** **Indirect effect slopes:** *B*=0.16 (95%CI 0.06 to 1.07), β=0.19	

					Items included: “How often do you feel satisfied with who you are?”<br><br>Items measured on a 6-point scale, from 1 (never) to 6 (daily). Items reverse coded. Higher scores = low self-esteem.<br><br>***Coping:*** Single-item questionnaire.<br><br>Item asked “How often do you feel that you are capable of coping with most of your problems?” Responses ranged from 1 (never) to 6 (daily).		

					Item reverse coded. Higher scores = poor coping ability.		
**Social media use**							
Vernon, 2017	Australia<br><br>Longitudinal cohort study<br><br>Follow-up: 2 years	N=874<br><br>Age: range not reported<br>Baseline mean age: 14.4 years (SD not reported)<br><br>Sex: 59% female<br><br>Ethnicity: 57.2% were Caucasian, 7.2% Asian, and 1.6% Aboriginal or Torres Strait Islander, 23.3% other	***Social media use:*** Assessed using the problematic use of social networking scale.<br><br>Scale consisted of 4 items, which measured the degree to which adolescents invest emotionally in social networking:<br><br>1. "I prefer to spend time on Facebook/ Myspace/ Bebo rather than attend social activities/ events".<br>2. "I use Facebook/Myspace/Bebo as a way of making me feel good".<br>3. "I get into arguments with other people about the amount of time I spend on Facebook/ Myspace/ Bebo."<br>4. Item 4: "If I can't access Facebook/ Myspace/Bebo, I feel moody and irritable".	***Sleep quality***: Items, adapted from the School Sleep Habits Survey, asked:<br><br>During the during the previous<br><br>2 weeks, how often have you:<br><br>"felt tired or sleepy during the day"; "had an extremely hard time falling asleep"; "had a good night's sleep (reversed)"; "felt satisfied with your sleep" (reversed).<br><br>Response option were 1 (never), 2 (once), 3 (twice), 4 (several times),	***Depressed mood:*** Questionnaire from Michigan Study of Adolescent Life Transitions, comprising mean of 5 items.<br><br>Items included: "How often do you feel there is nothing nice you can look forward to; feel unhappy, sad, or depressed?"<br><br>Responses ranged from 1 (never) to 6 (daily). Higher scores = worse outcome	*Latent Growth Curve Mediation Models (Mediator: sleep disruptions; Covariate: gender, SES, pubertal timing).*<br><br>**Depressed mood: Indirect effect slopes:**<br>B= 0.181 (95%CI 0.132 to 0.244)<br><br>***Externalising behaviour:*** **Indirect effect slopes:**<br>B= 0.034 (95%CI 0.006 to 0.131)	Adolescents who increasingly invested in social networking reported increased depressed mood.<br><br>53% of this association was explained by increased sleep disruptions.<br><br>Increased investment in social networking was associated with increased externalizing behaviour.

			Responses ranged from 0= no social media profile, 1= completely disagree, to 5= completely agree.	and 5 (every day/night). Higher scores = poorer sleep quality.	***Externalising behaviour:*** Questionnaire comprising mean of 7 items. Items included: "In the past 6 months how often have you skipped school without parent permission?; how often have you gotten in a physical fight with another person?"<br><br>Items measured on 8-point scale from 1 (none) to 8 (31 or more times), Higher scores = worse outcome.		13% of this association was explained by increased sleep disruptions.
Viner et al., 2019	England<br><br>Longitudinal<br><br>Wave 1 = 2013<br>Wave 2 = 2014<br>Wave 3 = 2015	N=<br>Wave 1 = 12,866<br>Wave 2 = 10,963<br>Wave 3 = 9,797<br><br>Age:	***Social media use:*** Young people reported the frequency with which they habitually accessed or checked social media. Social media defined as any of the major networks (eg.	***Sleep adequacy:*** At wave 2, young people were asked to report their usual weekday bed-time and	***Mental health and wellbeing*** Wave 2: young people completed the 12-item General Health	*Analysed separately for boys and girls. Multinomial logistic regression*	Among boys, sleep as a single mediator was responsible for 4.8% of the effect of very

		Wave 1: 13–14 years<br>Wave 2: 14–15<br>Wave 3: 15–16<br><br>Sex:<br>Wave 1: 50% female<br>Wave 2: 50% female<br>Wave 3: 51.3% female	Facebook, Twitter, or Instagram), instant messaging or photo-sharing services (eg. WhatsApp, Blackberry Messenger, Snapchat, or Tumblr), among others. Example sites were quoted and updated at each wave.<br><br>Frequency of use was reported as never, weekly, every few days, daily, 2-3 times per day, or multiple (ie. more than three) times daily.	wakening time during the past month, and their duration of sleep (assumed as entire period between bed time and wake time).	Questionnaire (GHQ12).<br><br>Scale scores added and dichotomised at threshold of 3, high scores (3 or higher) indicative of psychological distress and probably psychiatric caseness<br><br>Wave 3: four questions on personal wellbeing, drawn from Office for National Statistics wellbeing surveys: i) “overall, how satisfied are you with your life nowadays?” ii) “overall, to what extent do you feel the	*adjusted for potential mediators (cyberbullying, sleep adequacy and physical activity)*<br><br>*Model 1: sleep as single mediator*<br>***<u>GHQ12 high score</u>***<br>***Boys:***<br>**Less than 8h sleep**<br>OR = 1.45 (95% CI 91.17-1.80), p=0.0006<br>Proportion mediated 4.8%<br><br>***Girls:***<br>**Less than 8h sleep** OR = 2.00 (95% CI 1.68-2.38) p<0.0001<br>Proportion mediated 17%	frequent social media use on later mental health.<br><br>Among girls, sleep as a single mediator accounted for 17% of the effect of very frequent social media use on later mental health.<br><br>Amongst girls only, sleep as a single mediator accounted for 33.9% of the effect of very frequent social media use on later life satisfaction.

					things you do in your life are worthwhile?" iii) "overall, how happy did you feel yesterday?"; and iv) "overall, how anxious did you feel yesterday?<br><br>Each question answered with a score from 0 (minimal) to 10 (high).<br><br>Each question used as a separate outcome	***<u>Wave 3 wellbeing</u>:***<br>*Ordinal logistic regression adjusted for potential mediators*<br><br>*Results for girls only*<br><br>***Life satisfaction***<br>*Model 1: sleep as single mediator*<br>***Girls:***<br>**Less than 8h sleep**<br>OR = 0.57 (95% CI 0.51-0.65), p<0.001<br>Proportion mediated<br>Sleep 33.9%<br><br>***Happiness***<br>*Model 1: sleep as single mediator*<br>***Girls:***	Amongst girls only, sleep as a single mediator accounted for 22.3% of the effect of very frequent social media use on later happiness.<br><br>Amongst girls only, sleep as a single mediator accounted for 12% of the effect of very frequent social media use on later anxiety.

						**Less than 8h sleep**<br>OR = 0.66 (95% CI 0.58-0.76), p<0.001<br>Proportion mediated<br>Sleep 22.3%<br><br>***Anxiety***<br>*Model 1: sleep as single mediator*<br>***Girls:***<br>**Less than 8h sleep**<br>OR = 1.35 (95% CI 1.18-1.54), p<0.001<br>Proportion mediated<br>Sleep 12%	
**Adverse implications of IED use -** *IED overuse or problematic use*							
Kwon et al., 2020	USA<br><br>Prospective longitudinal cohort study	N=801<br><br>Age: 13-15<br><br>Mean age: 14.45 (SD 0.85)	***Cybervictimisation:*** Used cyber victimisation scale to assess online bullying and sexual harassment online.	***Sleep quality:*** Adapted version of the Pittsburgh Sleep Quality Index (PSQI). Self-rated	***Depressive symptoms:*** Used revised Center for Epidemiologic Studies	*Hayes' (2017) process approach to dealing with the mediation model*	No direct association was observed between cyber-victimisation

	Follow-up: 6 months	Sex: 57% female<br><br>Ethnicity: 81% European American, 12% African American, 7% Hispanic/Latino, 4% multiracial, 1% Asian American, and <1% Native American	Scale consisted of 9 items. 6 items from Ybarra et al (2007), and 3 additional investigator-developed items:<br>1. Someone made me feel worried or threatened because they were bothering or harassing me online.<br>2. Someone threatened or embarrassed me by posting or sending messages/pictures of me online for others to see.<br>3. Someone sent me a sexual picture or video online that I did not want.<br><br>Respondents reported on the frequency of each item occurring within the past 6 months. Scores ranged from 1 (never happened) to 7 (every day or almost every day). Higher values indicated higher levels of cyber victimisation.	questionnaire assessing sleep quality and disturbances over 1 month. Modifications for adolescents by asking about sleep on school days and non-school days, and item regarding bed partners/room mates was removed. Two items assessing sleep disturbances due to noises from cell phones or other electronic devices were added.<br><br>Total PSQI score of >5 considered indication of clinically significant sleep disturbance or poor sleep quality.	Depression Scale Revised (CESD-R).<br><br>Scale comprised of 10-items which assessed depressive symptoms in the past week. Items include depressive affect, somatic symptoms, and positive affect.<br><br>Total scores ranged from 0 (rarely or none of the time) to 3 (all of the time).<br><br>Higher scores suggested greater severity of symptoms.	*Controlled for age, sex and depressive symptoms at baseline*<br><br>Indirect effect a*b = 0.039 (0.116) = 0.0045)	and depressive symptoms.<br><br>A significant indirect effect was identified from poor sleep quality on the relationship between cyber-victimisation and depressive symptoms. The mechanism of being depressed among adolescents who were cyber-victimised was better explained by the mediational pathway of poor sleep quality

Supplementary Table 5. Risk of Bias rating for individual studies assessing sleep outcomes

Included study IDs	Selection bias	Performance bias	Detection bias	Attrition bias	Selective reporting bias
Barber & Santuzzi, 2017	High	High	Unclear	High	Unclear
Bartel et al., 2019	High	Unclear	High	Unclear	High
Chang et al., 2022	Low	Low	Unclear	Unclear	Low
Foerster et al., 2019	Unclear	High	High	Unclear	Low
Garett et al., 2018	High	Low	High	Unclear	Unclear
Gumport et al., 2021	Unclear	Low	Low	Unclear	Low
Hamilton et al., 2020	High	Unclear	High	Unclear	Low
Harbard et al., 2016	High	Unclear	Low	Low	Low
Heath et al., 2014	Unclear	Unclear	Low	Low	Unclear
Herge et al., 2016	Unclear	Unclear	Unclear	Low	Low
Jose, 2018	Low	Unclear	High	Low	Unclear
Kemp et al., 2020	Low	Unclear	Unclear	Low	Low
Kojima et al., 2019	Low	Low	Unclear	Unclear	Low
Lee et al., 2017	Low	High	Unclear	Unclear	Low
Maksniemi et al., 2022	High	Low	High	Low	Low
McManus et al., 2020	High	Unclear	Low	Low	Low
Patte, 2017	Unclear	High	High	Low	Unclear
Perrault et al., 2019	Low	Unclear	Low	High	Unclear
Poulain et al., 2019	Unclear	Unclear	Unclear	Unclear	Low
Schweizer et al., 2017	Low	High	High	High	Unclear
van der Schuur et al., 2019	Unclear	Low	High	High	Low
Vernon, 2017	Unclear	High	High	Unclear	Unclear
Vernon et al., 2018	Unclear	High	High	Low	Unclear
Werner-Seidler et al., 2019	High	High	High	High	Unclear

Yoo, 2020a	Low	Unclear	High	High	Low
Yoo, 2020b	Low	Unclear	High	Unclear	Low

Supplementary Table 6. Risk of Bias rating for individual studies assessing mental health outcomes mediated by sleep measures

Included study IDs	Selection bias	Performance bias	Detection bias	Attrition bias	Selective reporting bias
Kwon et al., 2020	High	Unclear	Unclear	Low	Low
Vernon et al., 2018	Unclear	High	Low	Low	Unclear
Vernon, 2017	Unclear	Unclear	Low	Unclear	Unclear
Viner et al., 2019	Low	High	Low	High	Low
